# The Demographic Causes of European Sub-National Population Declines

**DOI:** 10.1007/s10680-025-09730-0

**Published:** 2025-04-03

**Authors:** Niall Newsham, Francisco Rowe

**Affiliations:** Geographic Data Science Lab, Department of Geography and Planning, University of Liverpool, Liverpool, UK

**Keywords:** Depopulation, Europe, Fertility, Mortality, Migration

## Abstract

Population decline is now established at the continental scale in Europe, occurring in abundance across sub-national areas and outweighing population growth. This represents an unfamiliar pathway of demographic change and is set to present unique challenges to the functioning of societies and economies. The nature of these challenges will be influenced by the demographic cause of population decline. Typically, low fertility is cited as the primary instigator, though it remains unclear of the ways in which unique interactions between fertility, mortality and migration have shaped contemporary population decline outcomes. This study empirically analyses the demographic causes of population decline in 732 sub-national areas extending across 33 European countries. Drawing on data derived from national statistics from 2000–2018, we employ a novel methodological approach consisting of decomposition, multivariate functional principal component analysis, and k-medoid clustering to identify the dominant demographic processes underpinning European depopulation. Our analysis reveals five unique signatures, encoding nuanced contributions from fertility, mortality and migration changes. Population decline is found to be a multi-causal process, with natural deficits and negative rates of net-migration both operating depopulations in most instances. We conclude that natural deficits are ubiquitous in causing sub-national population declines with net-migration patterns responsible for determining annual rates of population loss. We model the relationship between these signatures and wider demographic, socio-economic and geo-spatial attributes, finding that a distinct combination of contextual factors are associated with different demographic causes of population decline.

## Introduction

A longstanding trajectory of population growth has reversed in Europe, with population decline prevailing as the new order of continental population change. Population decline is set to define twenty-first century European population dynamics, with depopulation expected to persist and shrink the population of Europe by 157 million, or 21%, by 2100 (UN, 2022). Alongside reductions in absolute population size, population decline is also associated with changes in the demographic composition and social, cultural and economic characteristics of populations (Franklin, 2020; Bock & Haartsen, 2021). A series of novel challenges are likely to arise as a result of such unfamiliar demographic change, relating to the economic and social functioning of affected areas. Namely, depopulation threatens economic productivity and growth (Coleman & Rowthorn, 2011), signals shrinking tax revenues effecting the funding of public services (Van Dalen & Henkens, 2011), and diminishes the competitiveness of areas whilst suppressing investment (Elshof et al., 2014) and immigration (Franklin, 2019). Furthermore, changing population compositions under depopulation may result in reduced diversity (Franklin, 2020), increased inequality (Bellman et al., 2018) and social isolation through the erosion of community networks (Hospers & Reverda, 2015). Importantly, the nature of challenges associated with population decline will depend on the demographic cause of population decline. Different depopulation processes have distinct effects on changing age structures, for example, which present unique challenges.

The origins of population declines are typically attributed to low fertility rates (Reher, 2007; Lutz & Gailey, 2020), with the wider demographic context seldom considered. As such, little is known about how contemporary demographic processes are driving population declines across Europe. The heterogeneous nature of depopulation trajectories (Newsham & Rowe, 2022) suggest that unique interactions between fertility, mortality and migration outcomes are operating across the continent. Broad differences exist amongst European regions regarding demographic, social, cultural, economic, political, and environmental configurations (Bongaarts, 1978; Campisi et al., 2020; Cutler et al., 2006; Rowe et al., 2019), though their influence on population decline processes are yet to be assessed. An enhanced understanding of the differential demographic causes of European population declines are crucial to devising effective strategies to combat population declines through addressing the root demographic cause of population decline and not just its consequences (European Parliament, 2021). Understanding the differential drivers of population decline is also important for the development of demographic theory and to inform the production of population forecasts, both of which will be crucial to the management of future population loss.

Much of academic and political discourse focuses on national scale population declines, yet it is inherently a localised occurrence. Across Europe, population declines are regularly measured in quantifications of regional, municipal and neighbourhood population size, even within countries experiencing population growth (Franklin, 2020), and encompassing rural and urban areas alike (Raugze et al., 2017; Wolff & Weichmann, 2018). In this study, we place a focus on understanding sub-national population decline dynamics. Our research questions are twofold. We first set out to assess the dominant demographic causes of population decline across Europe, evaluating the contributions of fertility, mortality and net-migration to annual population changes. We then explore the extent to which causal processes are associated with demographic, socio-economic and geo-spatial characteristics of areas. The remainder of this paper is structured as follows. We begin by establishing demographic trends across Europe, and outline their sensitivity to wider factors. This is accompanied by a review of the drivers of sub-national population decline. We continue with an explanation of the novel methodology used to study the different demographic contributions to European population declines. Next, we introduce the five depopulation signatures and describe their geographic distribution before analysing their demographic, socio-economic and geo-spatial configurations. Finally, we summarise our main contributions to the understanding of contemporary population decline causes.

## Literature Review

Population change is a function of fertility, mortality, and migration outcomes (Preston et al., 2000), and results in a decline when the net balance of these demographic processes is negative. Historically, interactions between these demographic drivers have resulted in population growth, with exceptional events such as war, disease or famine triggering temporary declines in population (Reher, 2005; Lutz & Gailey, 2020). Rapid population growth was observed as countries underwent successive transitions from high to low mortality and fertility rates, as described by the demographic transition model (DTM) (see Kirk, 1996). The significance of the DTM extends beyond describing historical demographic processes. It also established requisite demographic conditions for future population decline, including older age profiles and low fertility (Bongaarts, 2009; Reher 2007). Next, we summarise the general demographic trends across European sub-regions to contextualise the causes of sub-national population declines.

### Fertility

European nations are united in their observance of sub-replacement period fertility rates, below 2.1 births per woman. Low fertility has persisted since its onset in all European nations (UN, 2022), acting to diminish the size of successive birth cohorts and contributing to natural population decline. Low period fertility of late is explained by the postponement phenomenon (Kohler et al., 2006; Sobotka, 2004), in which union formation and fertility are delayed to higher reproductive ages, and ultimately reducing the extent of completed fertility outcomes (Lesthaeghe & Willems, 1999).

Though all nations are implicated by similar societal changes, differential rates of low fertility are observed across the continent. Nations experiencing population decline are distinguished by low fertility extremes, often below 1.5 births per woman. Lowest fertility rates are present across eastern and southern Europe (UN, 2022), reflecting the prevalence of population decline in these regions. In contrast, higher fertility is observed within more economically affluent nations of Northern and Western Europe, where population declines are more sporadic (Newsham & Rowe, 2022). Within-country fertility differentials are also prevalent across Europe, with most countries observing higher fertility rates in rural areas than in urban areas (Kulu, 2013).

Explaining fertility differentials in Europe, studies have uncovered nuanced social, cultural, and economic regularities influencing fertility outcomes. Delayed domestic independence is cited as a cause of low fertility extremes in southern Europe (Beaujouan, 2020; Reher, 1998). Gender equality (see McDonald, 2000), in which equal gender division of household labour reduces the burden of work-family conflicts and leads to positive fertility outcomes (Cooke, 2009; Myrskylä et al., 2011; Thomas et al., 2022). Such equality is greatest in northern Europe (Fisher & Robinson, 2011), where fertility is highest in the continent (UN, 2022). Relative successes of service-oriented policy measures that reduce the opportunity cost of children, particularly in northern and western Europe (Neyer & Andersson, 2008), as opposed to relative failures of income-support policies (Sobotka et al., 2019). Finally, increases in fertility rates are frequently observed as a result of higher fertility outcomes of migrant groups (Newsham & Rowe, 2021; Sobotka, 2008). However, such increases act to disproportionately benefit certain northern and western European countries where immigration is greatest (King & Okólski, 2018).

### Mortality

Mortality represents the primary way in which populations shrink and is therefore fundamental to population decline. From 2000 to 2018, life expectancy has increased in every European territory signalling longevity improvements that are conducive to population growth. However, 29 of 45 countries (64%) have recorded increases in crude mortality rates during the same period (UN, 2022), acting to promote natural population declines. Such increases are most prevalent across south, south-eastern, and central regions of the continent where mean ages are highest (UN, 2022). The increase in crude mortality rates may be partially explained by population ageing in Europe. Given the relationship between age and the force of mortality (Gompertz, 1825), population ageing is strongly associated with increases in crude mortality rates (Cheng et al., 2020).

As with fertility, differential trends in mortality are observed across Europe resulting from differences in the determinants of health relating to environmental, cultural, social and economic conditions, as well as public health policies and strategies (Cutler et al., 2006; Karanikolos et al., 2017). There exists a vast and persistent East–West European divide, with mortality higher in eastern and central Europe than elsewhere. This mortality gap is symptomatic of asynchronous health developments in which decreases in cardiovascular mortality were fundamental (Vallin & Mesle, 2004). As western European countries underwent decreased cardiovascular related mortality and improved longevity (Meslé et al., 2002), life expectancy stagnated in the East due to increased cardiovascular (Grigoriev et al., 2010) and lifestyle attributable mortality relating to obesity, smoking, and drug abuse (Janssen et al., 2021). Though a convergence has occurred since cardiovascular related mortality has decreased in the East (Hrzic et al., 2020), continued improvements in the west, albeit at a slower pace (Raleigh, 2019), mean that extensive divisions persist. Differential mortality rates are also observed within countries, with rural populations typically experiencing higher mortality rates than their urban counterparts (Bremberg, 2020). However, evidence for this across Europe is sparse and complex, with recent research showing that rural–urban mortality disparities exist only amongst aged populations (Ebeling et al., 2022).

### Migration

Migration is regarded as the most crucial component of contemporary European population change dynamics (Sobotka & Fürnkranz-Prskawetz, 2020), though it is also the most volatile and uncertain, with measurement difficulties hindering its accurate quantification (Zagheni et al., 2014). Migration represents a unique demographic process, as it simultaneously contributes to both population growth in receiving areas, and population decline in sending areas. Its multifaceted effects have a profound influence on population redistribution and exacerbates differential population change trajectories (Rowe et al., 2019). This significance is most pertinent within Europe, where free-movement agreements facilitate international migration between European Union member states.

Intra-European migration flows favour select countries, with the UK, France, Germany, Switzerland, Italy and Spain receiving 75.4% of migrants since the 2004 European Union expansion (King & Okólski, 2018). In contrast, intra-European flows have contributed to depopulation in central and eastern countries, where significant out-migration has occurred, particularly within former-soviet states (Fihel & Okólski, 2019). When combined with trends of extra-European migration, which also disproportionately benefit the aforementioned nations (Eurostat, 2022), contemporary migration flows serve to bolster the population of a few countries whilst simultaneously contributing to depopulation in the rest. Similarly, contemporary internal-migration trends also serve to disproportionately benefit some sub-national regions. Most significant are rural to urban migration flows that have long persisted across Europe (Raugze et al., 2017), particularly amongst young and educated populations seeking greater economic opportunities in large cities (Rodríguez-Vignoli & Rowe, 2018). Processes of urban sprawl are also prevalent across Europe, with migration flows to peripheral metropolitan areas resulting in population growth, often at the expense of urban centres (Rowe et al., 2019).

The significance of migration is extended by its influence on fertility and mortality outcomes. Given that migration is most prevalent amongst young adults of peak reproductive ages, out-migration results in reduced fertility outcomes in origins as migrants undergo childbirth in destinations (Johnson & Lichter, 2019). Out-migration therefore represents a dual depopulating force in origin areas, as populations are immediately reduced and anticipated future fertility is lost. Regarding mortality, the young age profiles of migrants lead to a hollowing out of age structures and accelerates population ageing (Fihel et al., 2018). Given the positive association of ageing and increased mortality outcomes (Cheng et al., 2020), out-migration then represents an avenue in which crude mortality rates are exacerbated in places of origin. In contrast, in-migration produces the opposite effects and demonstrates its multifaceted potential in mitigating population declines in receiving areas.

### Drivers of Sub-National Population Decline

Though most academic and public discourse surrounding population decline concerns national level scales, depopulation is inherently a local process. It occurs within households, neighbourhoods and communities but is detectable only at aggregated units for which relevant data is collected. Localised instances of depopulation can be masked if situated within larger contexts of population growth (Franklin, 2019), rendering it difficult to recognise and manage. This is reflected by a sparsity of research into sub-national depopulation processes. Despite this, it is known that sub-national depopulation is a heterogeneous process, occurring at different intensities and timings across Europe (Wolff & Weichmann, 2018; Newsham & Rowe, 2022) and with various demographic, geographic and socio-economic characteristics mediating distinct demographic processes (González-Leonardo et al., 2023). Fertility, mortality and net-migration outcomes are dependent on area-specific influences from social, cultural, economic, political, and environmental determinants (Bongaarts, 1978; Campisi et al., 2020; Cutler et al., 2006). As a result, differential demographic drivers co-exist across space and time, operating to produce unequal developments in population change. Next, we briefly describe contextual characteristics associated with sub-national population decline.

Firstly, as a demographic process, population decline is dependent on the demographic composition of populations. Empirical analyses have determined that the size and structure of populations are the most important factors associated with trajectories of population decline (González-Leonardo et al., 2023; Johnson et al., 2015). Demographic events occur at various stages of the life course but follow rigid age-patterns. For example, younger populations are at a greater risk of experiencing fertility and migration outcomes, whilst elderly populations are at greater risk of mortality. Regarding population decline, smaller and older populations are linked to trajectories of persistent and rapid depopulation (*ibid*). The loss of young populations, primarily through migration, accelerates ageing which in turn contributes to depopulation (Reynaud & Miccoli, 2018; Rodríguez-Vignoli & Rowe, 2018). Additionally, the share of migrant populations is also a key determinant of population changes, with established migrant settlements reinforcing positive migration flows by attracting new immigrant populations (Patias et al., 2023). Across Europe, migrant populations and their descendants also tend to experience higher fertility outcomes than native populations (Kulu et al., 2017), contributing to higher fertility rates (Newsham & Rowe, 2021; Sobotka, 2008).

Secondly, the positioning of areas within the urban–rural hierarchy also dictates demographic trends. Rural and urban areas differ in population size, composition, density, and economic function and thus experience differing demographic outcomes. Rural areas are most prone to population decline and are more likely to experience persistent and fast-paced depopulation trajectories (Newsham & Rowe, 2022). Economic restructuring and technological advancements have diminished economic opportunity in rural areas, resulting in mass out-migrations of young populations and depopulation (Johnson & Lichter, 2019; Viñas, 2019). Though population decline is less prominent in urban areas (Newsham & Rowe, 2022), examples across Europe are abundant (Wolff & Weichmann, 2018). Causal explanations refer to self-propagating mechanisms involving place-specific processes of economic decline, out-migration, natural population loss and sub-urbanisation (Haase et al., 2016; Hartt, 2018; Hoekveld, 2012). Certain urban areas are, however, better aligned to resist depopulation, particularly those with more dynamic and vibrant economies (Rodríguez-Pose & Ketterer, 2012), larger in population (Wolff & Weichmann, 2018) and younger age structures (Haase et al., 2016).

Finally, a range of geographic factors are associated with population decline. Numerous studies have identified the role of location in determining the direction of population changes. Peripheral rural areas with poor physical and economic accessibility are most implicated by population decline (Li et al., 2019), whereas those in close proximity to urban areas benefit from population growth via sub-urbanisation and counter-urbanisation migration flows (Pužulis & Kūle, 2016). Territorial characteristics are also influential to population decline, with geographically isolated areas of high altitude prone to intense rates of depopulation (Reynaud et al., 2020; Viñas, 2019) and coastal regions more conducive to population growth (González-Leonardo et al., 2023). Economic performance of neighbouring areas can also influence migration trends, with economic growth in adjacent cities leading to urban decline in some European cities (Wolff & Weichmann, 2018).

## Data and Method

### Data

To study the demographic causes of European population decline, we collect annual demographic data for 2,035 sub-national areas in 43 countries between the years 2000 and 2018. Our data captures annual changes in population size, fertility, mortality, and net-migration outcomes. We build a spatial panel database consisting of Eurostat and national statistics data, see Appendix 1 for data sources by country. We make use of the NUTS (Nomenclature of Territorial Units for Statistics) regional classification system, collecting data for NUTS 3 units which represent small sub-national regions, though varying according to country-specific geographic definitions. For countries outside of the European Union, which are excluded from the NUTS classification system, these sub-national units also correspond to small-regions (see Appendix 2 for the number of units in our analysis by country). Given the large spatial scale of our study and the extent of demographic data, our time series is constrained because of the lack of data capturing population at small geographical scales. In some areas of Europe, population decline is an historic process (Martí-Henneberg, 2005), and we recognise that our data does not capture its onset in all areas. We also utilise national statistics data to study the contextual factors associated with distinct cause-of-decline processes. Unlike our demographic data, these are time-invariant due to their infrequent collection, though we recognise that they may shape or be shaped by changes in local population structures. The demographic features considered include the proportion of elderly population, proportion of foreign-born population and unemployment rate. In addition to these contextual variables, we also calculate the proximity of each area to the nearest large city (with population greater than 1 million), the proximity to the coast, and average altitude using elevation data from the European Environment Agency.

From our dataset, we identify a total of 732 sub-national areas that have experienced an overall reduction in population size between 2000 and 2018, or from the first to last data record in instances of missing data. We recognise that we omit some European areas that have experienced episodic population declines but overall population growth between 2000 and 2018. We focus our analysis on areas recording overall population decline during this period as our purpose is to better understand the demographic causes of population decline.

Our database contains some areas that are affected by a boundary change, presenting a significant challenge in obtaining a complete time series. We are able to mitigate the disruptive impact of this through harmonisation, enabled through Eurostat and National Statistic correspondence files which maintain historical records of these changes. Our harmonisation procedure involved only new areas created from merging two or more areas, with demographic count information available for these historic units used to sum to the new area. Areas in our database correspond to the latest boundaries at the time of data collection. A greater challenge is the acquisition of complete data for all demographic components. Though complete data are available for the majority of areas, a total of 276 (37.7%) were missing data for a single demographic component in a specific year and were thus incomplete. Most instances of missing data relate to migration, reflecting the insufficient quality of migration data (Bell et al., 2014). We are able to limit the impact of this by computing a total of 1,395 net-migration estimates across 199 areas using the demographic balancing equation (Preston et al., 2000), given complete population, fertility, and mortality estimates—see Appendix 2. Despite this, data scarcity does not allow for a complete time series for all areas. As a result, we are unable to analyse the causes of population decline consistently throughout our time series for all areas, though our results are robust to their inclusion.

### Method

**Fig. 1:**
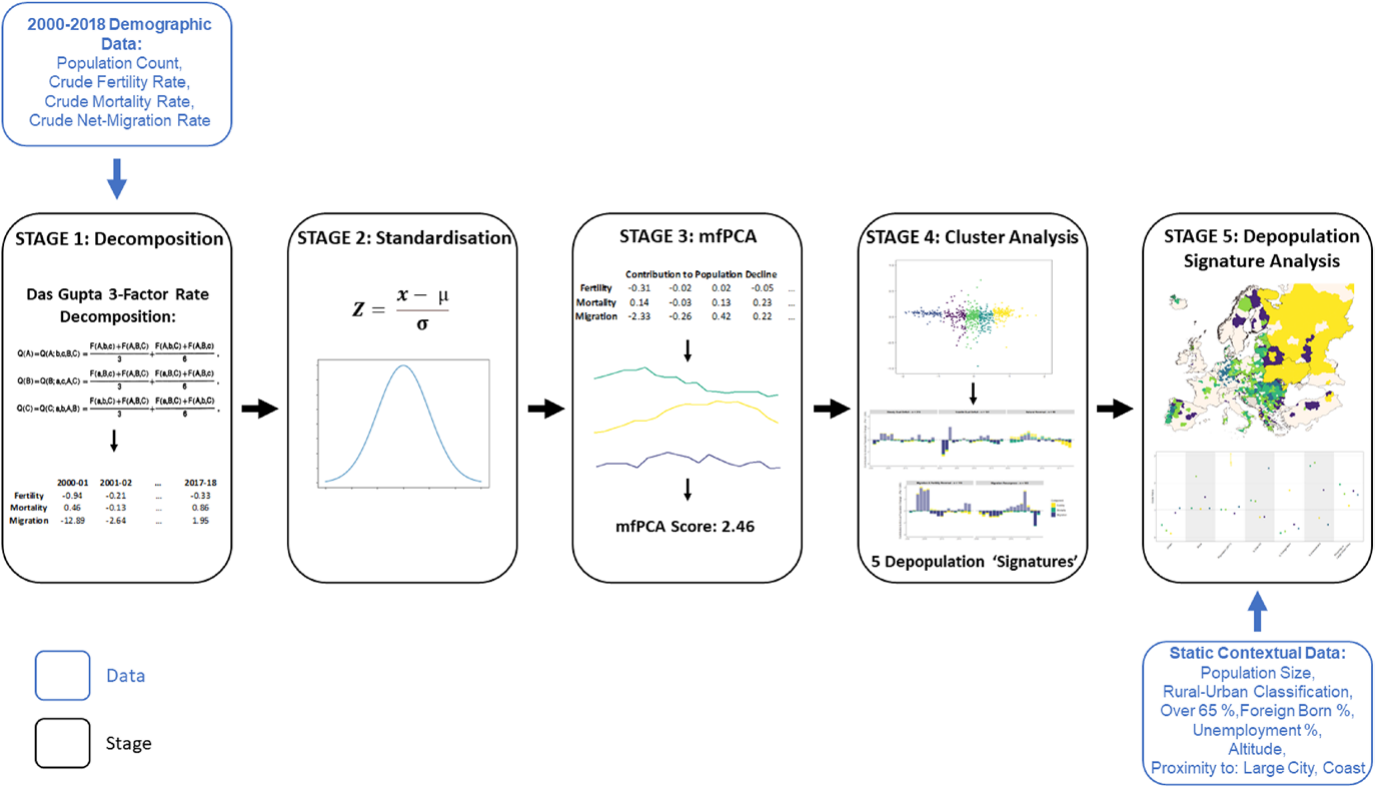
The five-stage method

We employ a novel five-stage methodology to analyse longitudinal patterns of demographic causes of population decline, outlined in Fig. 1. We first decompose changes in the annual rate of population decline to quantify contributions from fertility, mortality, and net-migration from 2000 to 2018. Next, we z-standardise each demographic variable independently to ensure a consistent scale for comparison of trends between areas. We then apply a functional data approach to measure the shapes of temporal trajectory curves representing the varying influences of each demographic driver on population decline. Fluctuations in crude rates of fertility, mortality and migration are captured empirically through Multivariate Functional Principal Component Analysis (MFPCA). Next, we apply a k-medoid clustering algorithm to group sub-national areas by similar temporal causes of population decline, referred to as depopulation signatures. Our use of the term signatures refers to the depiction of longitudinal demographic trends in the groupings that result from our cluster analysis. Finally, we analyse the demographic, economic, social, and geo-spatial attributes of areas to construct profiles of sub-national population decline across Europe and plot their geographic distribution.

#### Stage 1—Decomposition

In the first stage, we quantify the contributions of crude fertility, mortality, and net-migration rates to differing annual rates of population change from 2000–2018. To this end, we apply a three-factor rate decomposition technique (Gupta, 1993) to our longitudinal dataset, enabling the identification of time-specific demographic causes of population decline. Similar decomposition techniques have been used to disentangle the effects of demographic factors on changing rates of population growth (Horiuchi, 1992, 1995; Pullum & Jadhav, 2021; Vaupel & Canudas-Romo, 2002).

The demographic balancing Eq. (3) is embedded within the 3-factor rate decomposition formula (1,2), as presented in Gupta (1993, p. 21) and outlined below.

1 $$\begin{gathered} Q\left( a \right) = \frac{{F\left( {a,B,C} \right) + F\left( {a,b,c} \right)}}{3} + \frac{{F\left( {a, B, c} \right) + F\left( {a,b,C} \right)}}{6}, \hfill \\ Q\left( A \right) = \frac{{F\left( {A,b,c} \right) + F\left( {A,B,C} \right)}}{3} + \frac{{F\left( {A, b, C} \right) + F\left( {A,B,c} \right)}}{6}, \hfill \\ Q\left( b \right) = \frac{{F\left( {A,b,C} \right) + F\left( {a,b,c} \right)}}{3} + \frac{{F\left( {A, b, c} \right) + F\left( {a,b,C} \right)}}{6}, \hfill \\ Q\left( B \right) = \frac{{F\left( {a,B,c} \right) + F\left( {A,B,C} \right)}}{3} + \frac{{F\left( {a, B, C} \right) + F\left( {A,B,c} \right)}}{6}, \hfill \\ Q\left( c \right) = \frac{{F\left( {A,B,c} \right) + F\left( {a,b,c} \right)}}{3} + \frac{{F\left( {A, b, c} \right) + F\left( {a,B,c} \right)}}{6}, \hfill \\ Q\left( C \right) = \frac{{F\left( {a,b,C} \right) + F\left( {A,B,C} \right)}}{3} + \frac{{F\left( {a, B, C} \right) + F\left( {A,b,C} \right)}}{6}. \hfill \\ \end{gathered}$$

2 $$\begin{gathered} Fertility\,effect = Q\left( a \right) - Q\left( A \right), \hfill \\ Mortality\,effect = Q\left( b \right) - Q\left( B \right), \hfill \\ Net - Migration\,effect = Q\left( c \right) - Q\left( C \right) \hfill \\ \end{gathered}$$

where, *A* and *a* represent crude fertility in year t and t + 1, respectively. *B* and *b* represent crude mortality in year t and t + 1, respectively. *C* and *c* represent crude net-migration in year t and t + 1, respectively.

3 $$\begin{gathered} Pt = F\left( {A,B,C} \right) = A - B + C \hfill \\ Pt + 1 = F\left( {a,b,c} \right) = a - b + c \hfill \\ \end{gathered}$$

where the rate of population change (Pt) is equal to the crude birth (CBR) rate minus the crude death (CDR) rate plus the crude rate of net-migration (CNM).

The resulting output is a metric quantifying the effect size of each demographic component on the difference in the rate of population change between two consecutive years. Over a period spanning 19 years, we generate a total of 18 data points per component for each sub-national area representing the longitudinal contributions of fertility, mortality, and migration to changing rates of population decline.

#### Stage 2—Standardisation

Next, we rescale the decomposition data by z-standardisation to ensure a consistent scale for area-year observations and between demographic components. Areas in our sample differ widely in size and population numbers. Standardising the data ensures we can focus on the longitudinal trends and more easily compare population trajectories across areas.

4 $$Z = \varvec{ }\frac{{x - \varvec{ \mu }}}{{\upsigma }}$$

where $x$ is the original value, $\mu$ is the space–time mean value and $\sigma$ is the space–time standard deviation for all areas and years. We perform Z-standardisation for each demographic component separately, pooling together all areas of decline (*n* = 732) and years (*n* = 19).

#### Stage 3—Functional Data Analysis–Principal Component Analysis

In a third stage, we adopt a functional data analysis (FDA) framework to analyse the longitudinal effects of each demographic component on changing rates of population decline. FDA has been scarcely applied within the field of demography, and typically for forecasting (Hyndman & Booth, 2008; Shang et al., 2016). To our knowledge, FDA has not been used to identify groupings of areas with similar retrospective population change curves until now.

FDA contends with data presented as functions, defined as a series of observations occurring over a continuum. FDA considers the shape of a function, or curve, as its unit of analysis, as opposed to conventional multivariate data analysis which concern separate data points (Jung & Song, 2022; Ramsay & Dalzell, 1991). We use FDA here to analyse the shapes of demographic component curves and quantify differences and similarities in time-specific causes of population decline. This allows us to capture key differences in the time-dynamics of demographic components underpinning depopulation. For each area of decline (*n* = 732), we convert our standardised decomposition time-series data, derived from stages 1 and 2, into three functional data elements which represent the changing influence of fertility, mortality, and migration to rates of population decline. These functional data units are combined to create a multi-functional data unit for each area of decline. This process facilitates the application of multivariate functional principal component analysis (mfPCA). mfPCA reduces the dimensionality of our multi-functional fertility, mortality, and migration time-series data by extracting the key features that capture their variability over time. The variation in fertility, mortality, and migration curves are represented by functional principal component scores, and are used in subsequent trajectory clustering. Our application of mfPCA follows that of Happ and Greven (2018) and we use the R package ‘MFPCA’ for its implementation (see Happ-Kurz, 2020). The procedure of MFPCA in our study is described in four key steps and outlined below.

First, we perform univariate functional principal component analysis for each of the three functional data elements, resulting in estimated eigenfunctions and principal component scores for suitably chosen truncation lags, or principal components. Specifically, we obtain one principal component each for the fertility and migration data, whilst the mortality data identifies three principal components. We aim to select the minimum number of principal components that explain 90% of variance (see Ramsay & Silverman, 2005, chap 8.2). Subsequently, we conduct a multivariate functional principal component analysis using these two of these five total principal components, which together explain 100% of the variance across all functional data elements (Appendix 6). Second, all coefficients are combined into one matrix and a joint covariance matrix is calculated. This considers the covariation between each element by using the joint covariance of the principal component scores of all three elements. Next, eigenfunctions of the covariance matrix are found, with the first eigenfunction representing the greatest variation within the functional data and the last representing the smallest (Jung & Song, 2022). The final output is a set of mfPC scores which capture the functional characteristics of the demographic decomposition curves for fertility, mortality and migration, with similar scores demonstrating comparable characteristics in temporal cause of decline patterns.

#### Stage 4—Cluster Analysis

In the fourth stage of our methodology, k-medoid clustering is applied to the mfPC scores, enabling the grouping areas based on their shared longitudinal demographic causes of population decline. Here, medoids take the central data point of each cluster to minimise the sum of distances between all points in a cluster. We also explored the application of the k-means algorithm, though groupings were imbalanced in size due to its sensitivity to outliers. In our case, the k-medoid method results in a more accurate categorisation of areas by their demographic cause of depopulation. We judge the optimal number of clusters (*k*) both empirically and contextually, determining that five clusters are the most appropriate solution to strike a balance between detail and the identification of broad systematic patterns. A total of thirty empirical indices are tested, in accordance with the NbClust package (Charrad et al., 2014), with the majority confirming a five-cluster solution as optimal. Additionally, to further validate our decision, we conduct a sensitivity analysis to test the suitability of alternative k solutions. We find that a greater number of clusters (*k* > 5) presented recurrent cause of decline patterns and lower values (*k* < 5) masked nuanced causes, see Appendix 4.

#### Stage 5—Depopulation Signature Analysis

We analyse the composition of depopulation signatures, implementing a multinomial logistic regression model to understand the extent to which contextual attributes are associated with specific causes of population decline. We model a multicategorical outcome variable, representing our five cause-of-decline signatures, as a function of a series of demographic, socio-economic, and geographic independent variables. These include population size, rural–urban classification, proportion of elderly population, proportion of foreign-born population, unemployment rate, proximity to large city, proximity to the coast, and altitude. We standardise continuous variables, by z-standardisation, to allow for a robust comparison of coefficients. Our regression results are presented as odd ratios, enabling the assessment of the likelihood of an area experiencing a particular cause of population decline, given the status of each contextual attribute. We consider areas of population growth as a reference category, and therefore our results are interpreted as the likelihood of experiencing a specific cause of decline relative to population growth. In this final stage, we also explore the geographic distribution of causes of decline across Europe, discerning geographic regularities, and anomalies. We consider this, alongside results of our logistic regression to construct descriptive profiles of areas bound to a specific cause of population decline.

## Results and Discussion

We evaluate the extent to which differential demographic drivers have shaped contemporary population declines in two critical stages. Resulting from the initial four stages of our methodology, we first introduce five depopulation signatures which capture different demographic causes of sub-national population decline. We then analyse the contextual characteristics associated with each signature, quantifying differences in the demographic, socio-economic and geo-spatial configurations of areas to construct profiles of areas that experience a particular cause of population decline.

### Depopulation Signatures

**Fig. 2:**
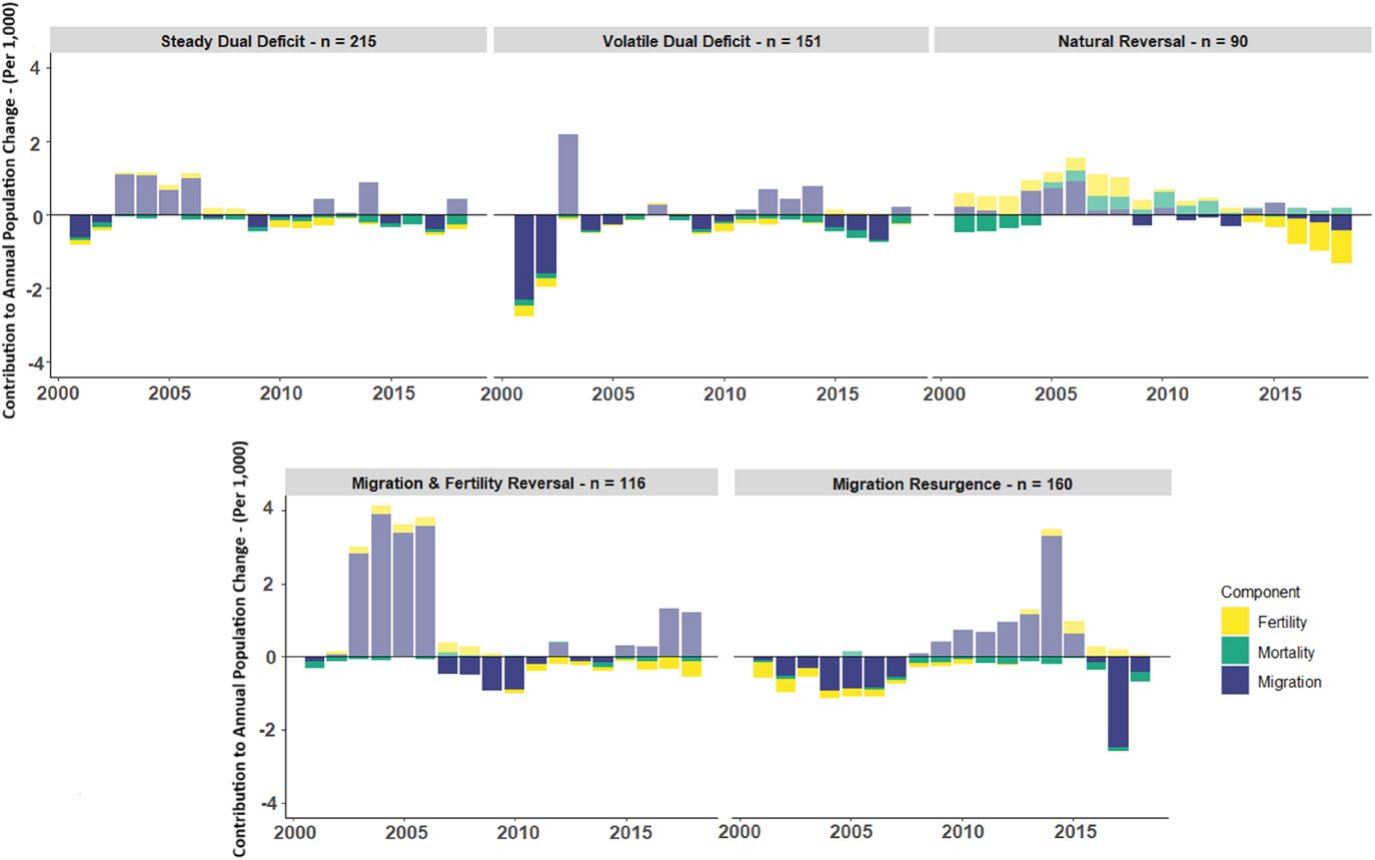
Depopulation signatures, depicting demographic contributions to annual rates of population change in areas of population decline

European sub-national areas follow differential trajectories of population decline, distinguished by differences in its pace, timing, and ordering (Newsham & Rowe, 2022). This suggests that differential demographic processes co-exist across the continent, and underpin such trajectories. In Fig. 2, we present five unique depopulation signatures that empirically represent the longitudinal contributions of fertility, mortality, and migration to population decline processes within European sub-national areas. Positive values represent an annual increase in contributions to population growth relative to the previous year. Neutral scores indicate no annual change and therefore no contribution to changing rates of population change for that period.^1^ Negative values indicate a contribution to population decline, and are emphasised in Fig. 2 to illustrate depopulation processes. We provide a contextualisation of our depopulation signatures in Appendix 3, presenting the trajectories of crude fertility, mortality and net-migration that are crafted by the annual changes depicted in our signatures.

Figure Depopulation signatures, depicting demographic contributions to annual rates of population change in areas of population decline.

#### Steady Dual Deficit

This signature depicts moderate contributions from all demographic drivers. Here, mortality contributions are consistent towards population decline, with fertility also becoming a factor from 2010. Net-migration fluctuates, making contributions to both decline and growth in different years. Its contributions towards decline are relatively small; yet total population decline is substantial amongst areas in this signature at 11.1% from 2000 to 2018 (Table 1). This suggests that pre-existing demographic conditions, which are not captured within our time series, are significant in establishing a rate of considerable annual population loss.

**Table 1:** Descriptive statistics of depopulation signatures

Signature	N	Population change	(%)	Births deaths	Natural change	Contribution (%)	Net-migration	Contribution (%)
Steady dual deficit	215	− 5,230,908	8.72	6,806,319 9,188,776	− 2,382,457	45.55	− 2,167,351	41.43
Volatile dual deficit	151	− 4,301,564	11.10	5,422,804 7,503,172	− 2,080,368	48.36	− 1,528,576	35.54
Natural reversal	90	− 13,869,153	9.99	26,427,584 37,792,185	− 11,364,600	81.94	− 1,961,923	14.15
Migration and fertility reversal	116	-3,049,773	8.45	5,565,269 7,126,080	− 1,560,811	51.18	− 859,236	28.17
Migration resurgence	160	− 2,500,480	7.30	5,534,911 7,214,921	− 1,680,010	67.19	− 515	0.02

#### Volatile Dual Deficit

This signature depicts comparable contributions from fertility and mortality, though is distinguished from *Steady Dual Deficit* by more intense and regular contributions from net-migration. This signature is bookended by significant contributions from migration, though natural change has the greatest contribution overall (Table 1).

#### Natural Reversal

This signature captures a reversal in the direction of contribution from fertility and mortality. Fertility initially contributes to population growth, though it gradually diminishes and eventually contributes to population decline. Conversely, mortality promotes depopulation until 2005, before it abruptly reverses to foster population growth. The significance of contribution from natural drivers is emphasised in Table 1, where 81.94% of population declines are accounted for by fertility and mortality changes.

#### Migration and Fertility Reversal

This signature is also characterised by a reversal in contribution, though instead concerning migration and fertility. Initial contributions to population growth from migration are reversed in 2006, whereby net-migration declines drive depopulations until 2015. This pattern is mirrored by fertility, shifting to a contribution to depopulation from 2007 until 2018. A relatively low overall contribution from net-migration (Table 1) suggests that the fertility reversal is most significant in causing population declines.

#### Migration Resurgence

This signature is characterised by a resurgence in positive net-migration contribution. Initially, migration contributes towards population decline, though later reverses to promote population growth until 2015. A sudden and considerable depopulation contribution is recorded in 2015, with negative net-migration resuming a trend to fuel depopulation. A similar shift in contribution from decline to growth is observed for fertility, though a resurgence in depopulation contribution is not observed. Meanwhile, mortality displays a gradually increasing contribution to depopulation. Natural demographic drivers are the main apparent cause of population decline (Table 1), given the significant positive net-migration upswing.

Taken together, our signatures represent the different demographic causes of European sub-national population decline. Despite their variability, they also depict some regularities. Most crucially, we observe similar contribution patterns from mortality and fertility between most of our signatures. All signatures, except *Migration Resurgence,* depict an increasing contribution to depopulation from fertility declines over time. The timing of such change is consistent, tending to occur from 2010 and perhaps reflecting a lagged impact of the Global Financial Crisis (Sobotka et al., 2011). This regularity supports the notion of a near-ubiquitous fertility decline in Europe resulting from rising unemployment and uncertainty (Vignoli et al., 2020; Matysiak et al., 2021), and demonstrates its effect on promoting population declines. A similar temporal regularity is observed for mortality, with a consistent moderate contribution to depopulation in all but one signature, *Natural Reversal.* These patterns demonstrate a commonality in depopulation processes amongst European sub-national areas, with natural change conditions near-ubiquitously driving contemporary population declines. Furthermore, our depopulation signatures illuminate the critical role of migration in depopulation processes. Unlike the contributions from fertility and mortality, net-migration contributions are less regular across signatures in both timing and magnitude. However, except for *Migration Resurgence,* all signatures demonstrate a notable contribution from migration (Table 1), indicating that population decline is a multi-causal process resulting from the cumulative or often counterbalancing impacts of fertility, mortality, and migration.

### Characteristics of Depopulation Signatures

**Fig. 3:**
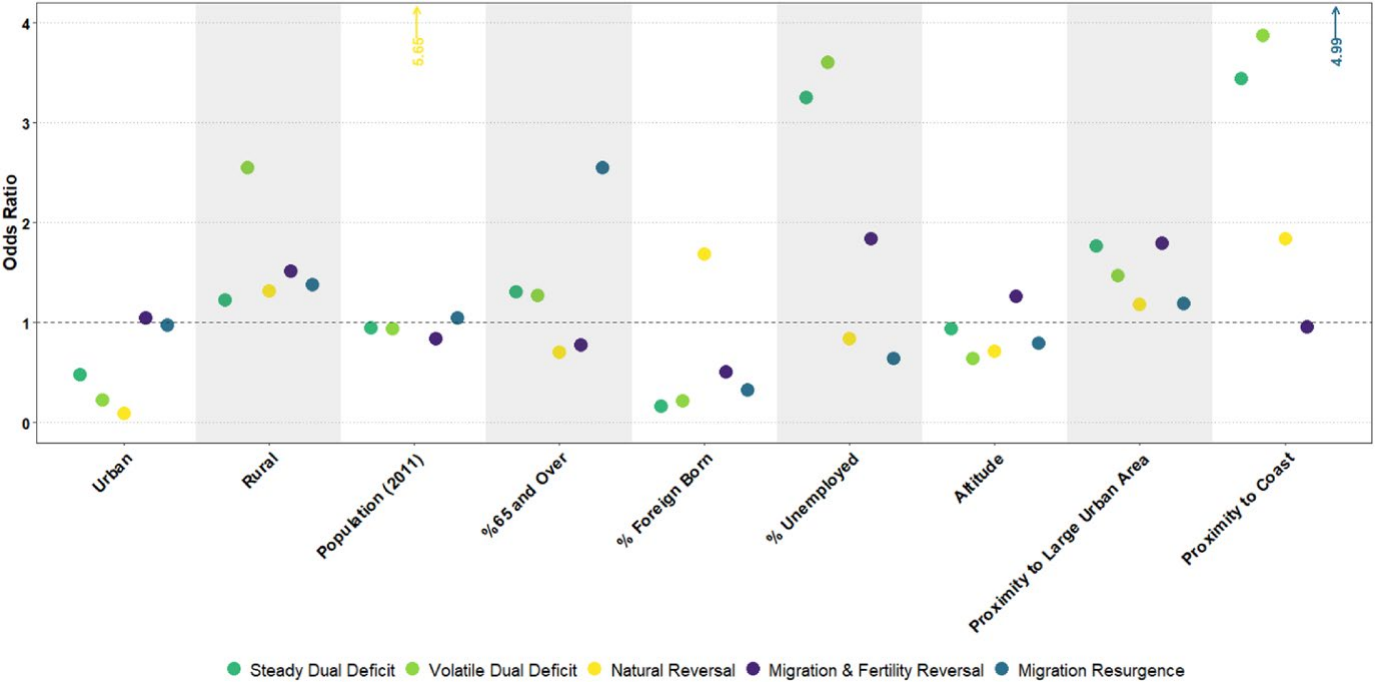
Multinomial logistic regression model

Next, we seek to understand the underpinning contextual features of our five depopulation signatures. To this end, we estimate a multinomial logistic regression model (Fig. 3) using our depopulation signatures as outcome variables and population growth as a reference category, with estimated coefficients presented as odds ratios. We interpret the results as the likelihood of an area experiencing a particular signature of population decline relative to population growth. Specifically, we examine the socio-economic, demographic, and geo-spatial characteristics of areas in 2011, that are associated with our demographic signatures. Whilst we recognise that these attributes may change over time as areas undergo depopulation (Franklin, 2020), data limitations prevent our ability to capture such temporal processes.

The results reveal key differences in the demographic, socio-economic and geo-spatial configurations of depopulation signatures. Firstly, the results indicate that rural areas are more likely to experience all cause-of-decline processes than intermediate areas (our reference category), particularly the *Volatile Dual Deficit* depopulation signature*.* Given that this signature depicts a contribution to depopulation from mortality rate increases (Fig. 2), this finding likely reflects the elder age profiles of rural areas (UNECE 2017) with a greater mortality risk (Cheng et al., 2020). Urban areas, on the other hand, show a lower odds ratio to experience population decline. In this context, if urban areas should experience decline, the results indicate that they are most likely to undergo *Migration & Fertility Reversal* and *Migration Resurgence* signatures. This signifies the propensity for urban revival, whereby decline-to-growth trajectories are enabled by positive net-migration flows Appendix 3. Urban areas act as centres for economic opportunity and therefore possess an ability to attract migrants (Rodríguez-Vignoli & Rowe, 2018; Zelinsky, 1971). Indeed, contemporary migration dynamics typically concern movements to urban areas, either from underperforming rural regions, or other urban areas (Rowe et al., 2019).

Our regression model also evidences how demographic attributes can influence causes of population decline. Here, we consider the proportion of elderly and foreign-born populations. The results show positive odds ratios for the percentage of population aged 65 and over for the signatures *Steady Dual Deficit, Volatile Dual Deficit, and Migration Resurgence*. Given that these signatures are characterised by increasing mortality rates (Appendix 3) and thus contributions to population decline (Fig. 2), the positive association likely captures the relationship between ageing and increased mortality risk (Cheng et al., 2020). Interestingly, we observe the strongest positive association between aged populations and the depopulation signature *Migration Resurgence*. This may reflect the retirement peak, whereby migration outcomes tend to increase amongst elderly populations (Rogers & Castro, 1981). Positive migration inflows to these areas may also be symptomatic of the high mortality outcomes that also characterise this signature. High mortality rates would act to increase the number of vacant properties, creating housing opportunities for migrants to replenish populations (Lycan & Rynerson, 2013). Regarding the extent of foreign-born populations, we find that all signatures display a negative association, except for *Natural Reversal.* With higher proportions of foreign-born populations then more associated with population growth, this result highlights the significance of positive international migration flows in reinforcing population growth (Patias, 2023). Furthermore, given the reversal of natural demographic conditions depicted in the signature *Natural Reversal* (See Figs. 2 and Appendix 3), the sole positive association here evidences a population growth effect outside of net-migration gains, namely in the form of fertility increases (see Sobotka, 2008; Newsham & Rowe, 2021) and mortality decreases (Aldridge et al., 2018; Domnich et al., 2012).

Examining the effects of unemployment, we also assess the role of economic opportunity in determining cause-of-decline processes. We find that unemployment is positively associated with some depopulation signatures, namely *Steady Dual Deficit, Volatile Dual Deficit, and Migration & Fertility Reversal.* These signatures depict either consistent negative or decreasing net-migration trends (Appendix 3) and a rural orientation (Fig. 5). This positive association then likely evidences the significance of poor economic opportunity in shaping negative migration outcomes of rural areas (Johnson & Lichter, 2019). Our results extend this, showing a positive association between unemployment and population decline.

Finally, our multinomial regression model illuminates the role of geo-spatial characteristics in determining population decline outcomes. Our results show a near-consistent negative effect between population decline outcomes and altitude, indicating that areas situated in higher altitudes are more likely to observe population growth than decline. Though this may differ in specific contexts as there is evidence from Italy and Spain pointing to a positive relationship between depopulation and altitude (González-Leonardo et al., 2023; Reynaud et al., 2020). We also explore the propensity for distinct depopulation processes in disconnected and less-desirable areas, represented by proximity to large urban areas and the coast. Our results show consistent positive odds ratios for both spatial variables, indicating that increased remoteness to large urban areas and coastal areas are associated with population decline processes. The effect of proximity to large urban areas is consistent for all depopulation signatures, though is volatile for coastal proximity. Depopulation signatures of *Steady Dual Deficit, Volatile Dual Deficit* and *Migration Resurgence* display particularly high odds ratio coefficients, whereby there is no association for the signature *Migration and Fertility Reversal*.

### Geographic Distribution of Depopulation Signatures

**Fig. 4:**
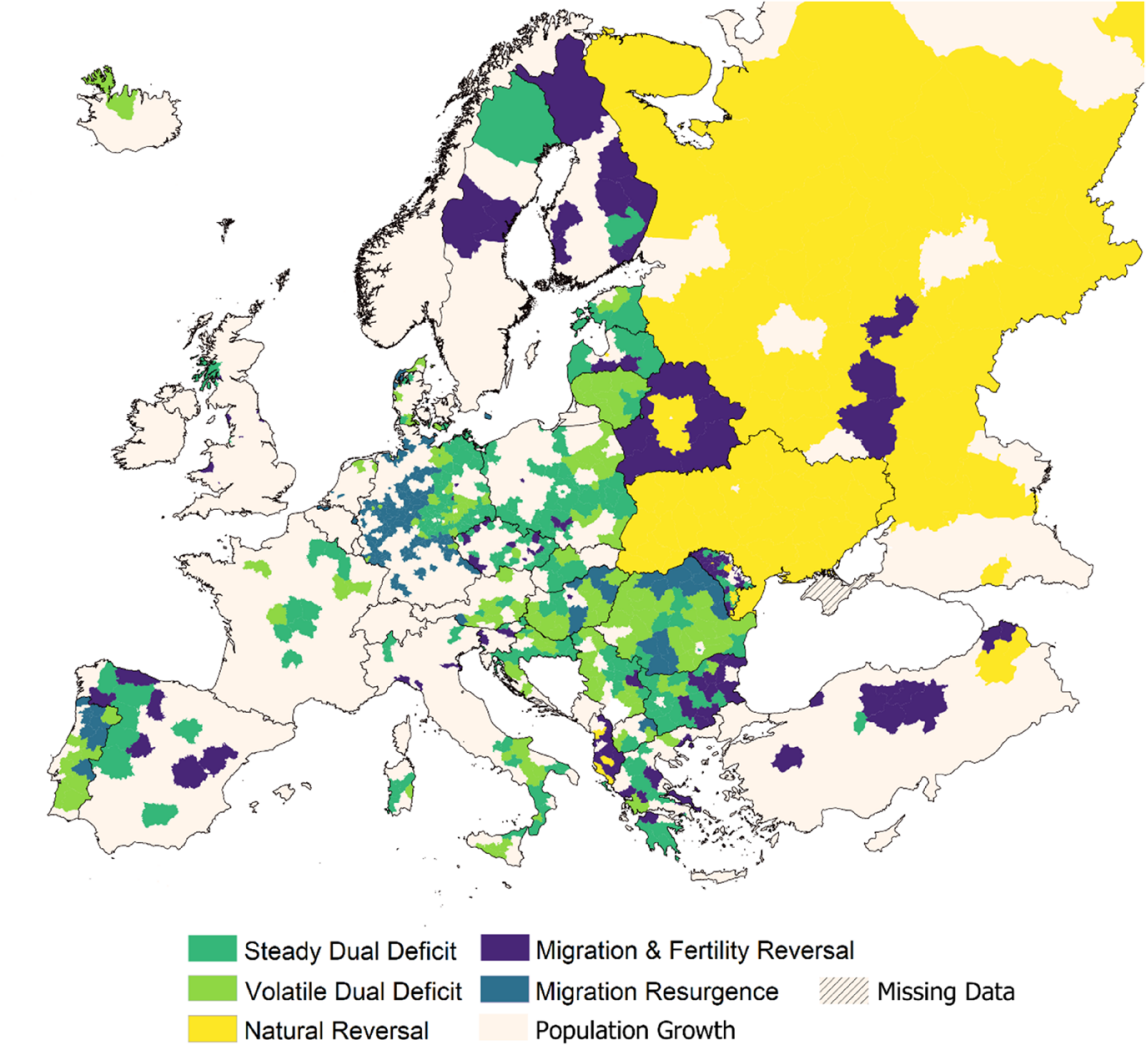
Geographic distribution of depopulation signatures, 2000–2018

Next, we explore the geographic distribution of cause-of-decline signatures and observe distinct within-country and cross-country spatial patterns. Figure 4 displays the geographic location of each cause-of-decline signature; areas that have observed a population growth are included to provide a holistic view of contemporary population change. First, we observe considerable heterogeneity in the geographic distribution of depopulation signatures across the continent. Particularly, we note how most signatures are represented in all European sub-regions, highlighting the complexity of demographic regimes across Europe. We also acknowledge this heterogeneity within countries, which is prevalent across south-eastern European nations but also considerably within Germany, Portugal, Belarus, and Hungary. We also observe some general geographic consistencies. Notably, regions in Eastern Europe are overrepresented by *Natural Reversal* processes, particularly within Russia, Ukraine, and Belarus. This signature of depopulation captures the influence of mortality rate decreases (Hrzic et al., 2020) and fertility rate increases (Romaniuk & Gladun, 2015; Shcherbakova, 2022), observed within these countries from 2005–2015, on decreasing the rate of population decline. Additionally, we observe how depopulation signatures that depict natural divergences, *Steady Dual Deficit* and *Volatile Dual Deficit,* are predominant in central and south-eastern European regions. The prevalence of these signatures here reflect the stalling of fertility and mortality rates that has plagued most of central and south-eastern Europe since the turn of the millennium (Sobotka & Fürnkranz-Prskawetz, 2020). We also detect the presence of the signature *Migration & Fertility Reversal* in peripheral areas throughout Europe, but particularly in the south-east (in Albania and Bulgaria) and east (in Belarus and Moldova) where out-migration processes are well documented (Denisenko et al., 2020). Finally, we observe the occurrence of *Migration Resurgence* processes in selective sub-national region, in western Germany and northern Hungary, Portugal and Romania. These trends are consistent with known regional migration disparities (see King & Okólski, 2018).

### Profiles of Depopulation Signatures

**Fig. 5:**
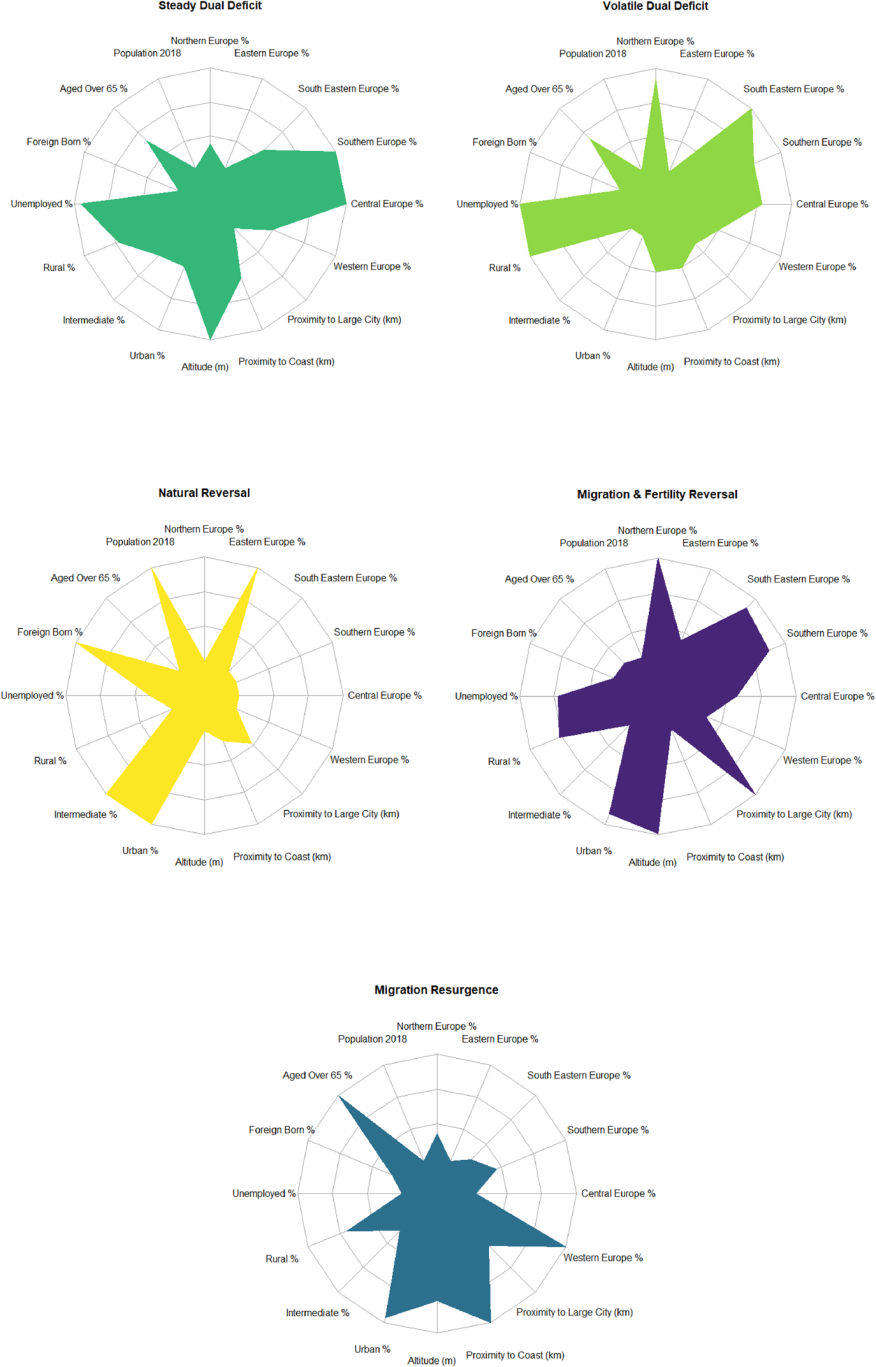
Contextual profiles of depopulation signatures

Synthesising the results of our analysis, we finally construct profiles of each depopulation signature to summarise their defining attributes (Fig. 5). Firstly, the signature *Steady Dual Deficit* consists of rural and intermediate areas with relatively small population sizes. Situated predominantly in in-land central and western Europe in high altitude regions, this signature is characterised by high unemployment rates, elderly and native populations. Similarly, the signature *Volatile Dual Deficit* also describes small population areas with high unemployment, elderly and native populations. Distinguishable characteristics are found within its greater rural orientation and low altitudes. This signature is well-distributed across Europe, and prevalent across the south-east, central, and western sub-regions. *Natural Reversal* predominantly consists of intermediate areas, but also is a relatively common urban depopulation signature. These areas are generally more populous, have the highest proportion of foreign-born populations and the lowest proportion of elderly. This signature is concentrated nearly exclusively within eastern European nations. The signature *Migration & Fertility Reversal* characterises high altitude areas isolated from large cities. This signature is well-represented across the rural–urban hierarchy and is prevalent in all European sub-regions. Finally, the signature *Migration Resurgence* is distinguished by its very high proportion of elderly, very low unemployment, and small average population size. This signature typically concerns in-land areas of western Europe.

## Conclusion

Population decline is becoming the dominant order of population change across Europe. This represents a significant shift in the demographic landscape of the continent and, in absence of social and technological reform, will pose unique threats to the functioning of societies. Such challenges will be determined by the severity of population loss, but also by its underlying demographic cause. Yet, we know little about how differential demographic drivers are producing population declines across the continent, rendering us largely unprepared. Understanding how changing fertility, mortality and migration outcomes are shaping trajectories of population decline is central to developing multiple avenues of research; for developing appropriate policy responses, population forecasting, and demographic theory. We present a methodological approach that adheres to the fundamental principles of demographic change to quantify the extent to which differential demographic processes have caused distinctive population declines. This is accomplished by applying novel functional data analysis, in which the shapes of demographic trajectory curves are compared to group areas undergoing population declines based on similar longitudinal causes.

Using publicly available data from Eurostat and national statistics institutions, we present evidence of the coexistence of distinct demographic causes of population decline. In total, we identify five unique depopulation signatures that are distinguished by nuanced longitudinal contributions from fertility, mortality, and migration. To summarise, these signatures capture processes of diverging and converging natural change, accelerating out-migration and negative-to-positive net-migration. Overall, we find that negative natural rates of natural change, that is rates of mortality outweighing fertility, is the most prevalent cause contemporary European population decline. These natural deficits are ubiquitous across all five signatures, but they do not act in isolation. In addition to natural deficits, most areas facing population losses also record population loss through migration. This depopulation is better characterised as a dual-cause outcome. Our evidence indicates that natural change is the most significant driver of population decline across Europe, though migration is most crucial in determining the pace of population decline within sub-national areas.

Our analysis also reveals how certain areas undergoing depopulation are predisposed to a particular demographic cause. We analyse a range of areal characteristics that are associated with each depopulation signature, capturing their demographic, socio-economic and geo-spatial configurations. We evidence how rural areas with particularly poor employment prospects and aged populations are predisposed to natural divergences, in which increasing mortality and falling fertility are exacerbating rates of population decline. We also find that urban areas, typically those smaller in size and isolated from chief cities and coastal areas, are associated with processes of migration resurgence and decelerating population decline. Additionally, we note that areas with a high proportion of foreign-born populations have observed a deceleration of population decline, owing to the simultaneous increase in fertility and decrease in mortality rates. Complementing this, we also present evidence of a well-defined geographic landscape of the way cause-of-decline processes are taking place in Europe. Population declines in eastern Europe are predominantly a consequence of natural deficits but are undergoing a deceleration reflecting a convergence process of increasing fertility and decreasing mortality. Depopulation is less prevalent in Western Europe, and negative net-migration balances have recently reversed to establish a force of population growth. In contrast, depopulation in central, southern, and particularly south-eastern European regions are a function of the combined effects of both natural deficits and negative net-migration outcomes. Coupled with sudden and large negative migration balances, increasing mortality and decreasing fertility have created a natural population deficit rapidly accelerating depopulation in these regions.

Our research is not without limitations. Firstly, our analyses are constrained by the scarcity of demographic data within countries of east and south-eastern Europe, the epicentres of European population decline. Particularly problematic are missing demographic data from initial years of our investigation and periodic migration estimates. Though we attempt to mitigate the impact through imputing net-migration estimates, we are unable to attain a complete time series for all demographic rates in all 732 areas of interest. Similarly, our data does not enable an investigation into the effects of changing age structures in producing population declines. The inclusion of age-structure information over time would provide a crucial level of understanding to the demographic causes of population decline, given the relationships between age and all demographic outcomes. Future studies should consider the inclusion of this information as critical. Though we acknowledge the role of age structure in demographic decomposition (see Canudas-Romo, 2003), we are unable to obtain relevant annual data for all European sub-national areas. Secondly, data limitations restrict our analysis to the years 2000–2018, meaning that we do not capture the onset of population decline in some places. We are also unaware of their historical demographic trajectories, and whether they have concerned an acceleration or deceleration of population decline. Furthermore, our data predates the COVID-19 pandemic, in which considerable disruption to demographic occurrences have occurred (see Sobotka et al., 2022; Wang et al., 2022; González-Leonardo et al., 2022). Finally, our research here has identified associations between a range of demographic, socio-economic and geo-spatial characteristics, and differential causes of population decline. However, it remains unclear whether such characteristics are responsible for instigating distinctive depopulation processes, or whether they are shaped as places undergo population decline. Future research should aim to disentangle these relationships, which would serve to illustrate a series of consequences that are seldom considered in research concerning depopulation (see Franklin, 2020).

Despite these limitations, our findings yield important advancements to knowledge of population decline processes and are particularly relevant in two key domains. Firstly, concerning policy formulation, given that demographic drivers of population decline fundamentally differ across different sub-national regions, we argue that mitigation initiatives must be tailored to individual areas and be devised with a focussed consideration of the specific underlying demographic cause. We argue that specificities of demographic causes will initiate unique challenges and thus require different solutions. Secondly, with regards to advancing population decline forecasts, the recognition of differential depopulation processes is crucial to devising accurate depictions of future population declines. Particularly, forecasts should explicitly consider the specific fertility, mortality, and migration contexts of areas, and perhaps include contextual information that has here been demonstrated to dictate depopulation processes.

## Figures and Tables

**Fig. 6:**
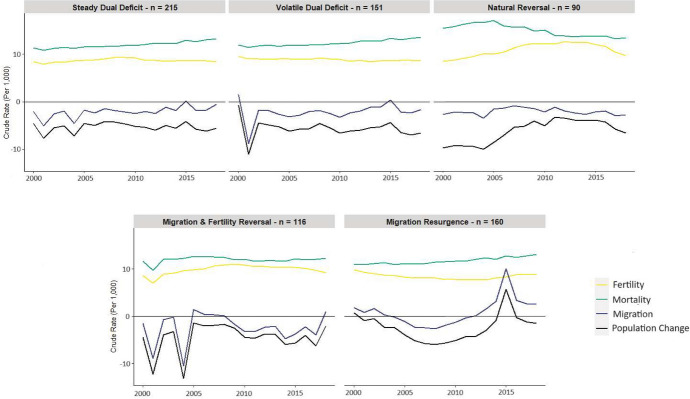
Demographic trends of depopulation signatures

**Fig. 7:**
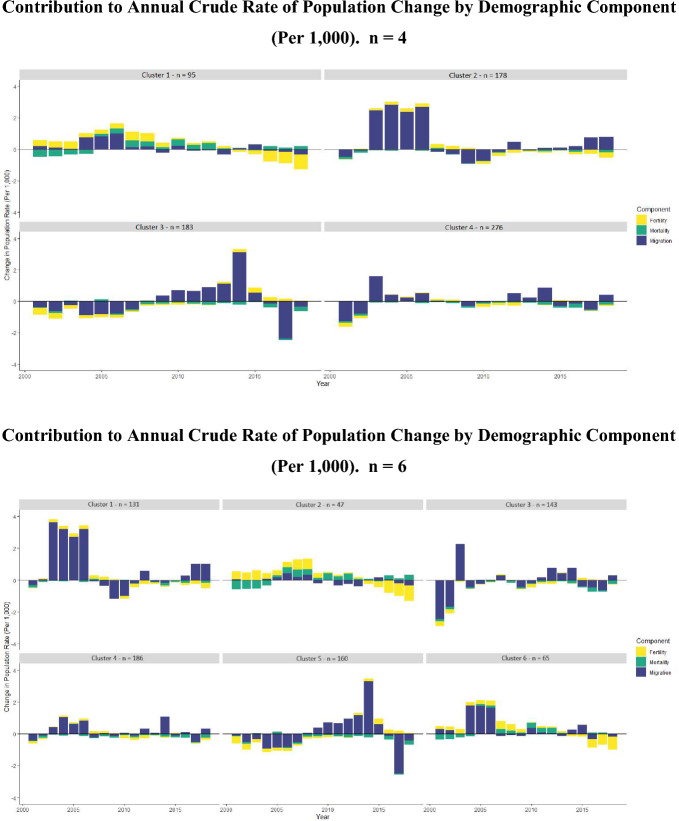
Sensitivity analysis. Results of k-medoid Clustering given k = 4 and k = 6

**Fig. 8:**
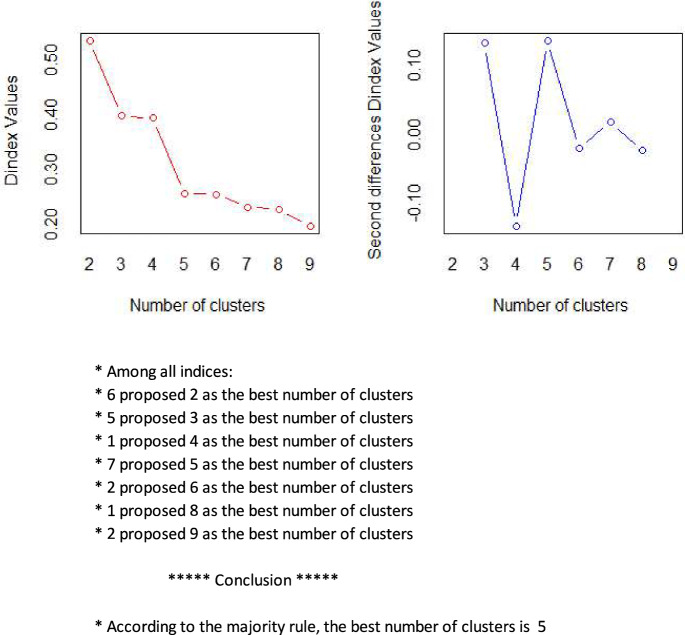
Optimal cluster solution evaluation. Outputs from the NbClust function

## Data Availability

The datasets analysed during the current study are available in the EUROSTAT demo_r_gind3 repository, https://doi.org/10.2908/DEMO_R_GIND3

## Appendix 1

See Table

**Table 2:** Data source by country and variable. Demographic counts includes annual population estimates, births, deaths, and net-migrations

Country	Variable				
	Demographic counts	Population over 65	Foreign born population	Unemployment rate	Rural–urban typology
Albania	National statistics	National statistics	National statistics	National statistics	Eurostat
Andorra	National statistics	National statistics	National statistics	National statistics	National statistics
Austria	Eurostat	Eurostat	National statistics	Eurostat	Eurostat
Belarus	National statistics	National statistics	National statistics	National statistics	National statistics
Belgium	Eurostat	National statistics	National statistics	Eurostat	Eurostat
Bulgaria	National statistics	Eurostat	National statistics	Eurostat	Eurostat
Croatia	Eurostat	Eurostat	National statistics	Eurostat	Eurostat
Cyprus	Eurostat	Eurostat	National statistics	Eurostat	Eurostat
Czechia	National statistics	National statistics	National statistics	National statistics	National statistics
Denmark	National statistics	National statistics	National statistics	National statistics	National statistics
Estonia	Eurostat	Eurostat	National statistics	Eurostat	Eurostat
Finland	Eurostat	Eurostat	National statistics	Eurostat	Eurostat
France	Eurostat	Eurostat	National statistics	Eurostat	Eurostat
Germany	National statistics	Eurostat	National statistics	Eurostat	Eurostat
Greece	Eurostat	Eurostat	National statistics	Eurostat	Eurostat
Hungary	Eurostat	Eurostat	National statistics	Eurostat	Eurostat
Iceland	National statistics	Eurostat	National statistics	National statistics	National statistics
Ireland	Eurostat	National statistics	National statistics	Eurostat	Eurostat
Italy	Eurostat	Eurostat	National statistics	Eurostat	Eurostat
Latvia	National statistics	Eurostat	National statistics	Eurostat	Eurostat
Liechtenstein	Eurostat	Eurostat	National statistics	Eurostat	Eurostat
Lithuania	Eurostat	Eurostat	National statistics	National statistics	Eurostat
Luxembourg	Eurostat	Eurostat	National statistics	Eurostat	Eurostat
Malta	Eurostat	Eurostat	-	Eurostat	Eurostat
Moldova	National statistics	National statistics	National statistics	National statistics	National statistics
Montenegro	Eurostat	Eurostat	-	Eurostat	Eurostat
Netherlands	National statistics	Eurostat	National statistics	Eurostat	Eurostat
North Macedonia	Eurostat	Eurostat	National statistics	Eurostat	Eurostat
Norway	National statistics	Eurostat	National statistics	National statistics	Eurostat
Poland	National statistics	National statistics	National statistics	Eurostat	Eurostat
Portugal	Eurostat	Eurostat	National statistics	Eurostat	Eurostat
Romania	Eurostat	Eurostat	National statistics	Eurostat	Eurostat
Russia	National statistics	National statistics	National statistics	National statistics	National statistics
San Marino	National statistics	Eurostat	-	-	National statistics
Serbia	National statistics	National statistics	National statistics	National statistics	Eurostat
Slova kia	National statistics	Eurostat	National statistics	National statistics	Eurostat
Slovenia	National statistics	Eurostat	National statistics	Eurostat	Eurostat
Spain	Eurostat	Eurostat	National statistics	National statistics	Eurostat
Sweden	Eurostat	Eurostat	National statistics	National statistics	Eurostat
Switzerland	Eurostat	Eurostat	National statistics	National statistics	Eurostat
Turkiye	Eurostat	Eurostat	National statistics	Eurostat	Eurostat
UK	National statistics	National statistics	National statistics	National statistics	National statistics
Ukraine	National statistics	National statistics	National statistics	National statistics	National statistics

2.

Our spatial panel dataset consists of annual population estimates and cross-sectional covariate data for a total of 2,035 areas across 43 European territories. However, as we place an explicit interest on studying population decline, we include in our analysis a total of 732 areas that are characterised by their absolute population loss from 2000 to 2018 (see Appendix 2 for included data by country). These data were sourced from Eurostat, and national statistics institutes. Eurostat data comprises of European Union member states only, though national statistics data is used in some instances where Eurostat data was incomplete.

## Appendix 2

See Table

**Table 3:** Proportion of complete annual demographic data by country (%)

Country	Country	n	2000	2001	2002	2003	2004	2005	2006	2007	2008	2009	2010	2011	2012	2013	2014	2015	2016	2017	2018
AL	Albania	10	0	0	0	0	0	0	0	0	0	0	0	100	100	100	100	100	100	100	100
AN	Andorra	1	100	100	100	100	100	100	100	100	100	100	100	100	100	100	100	100	100	100	0
AT	Austria	11	0	100	100	100	100	100	100	100	100	100	100	100	100	100	100	100	100	100	100
BG	Bulga ria	26	100	100	100	100	100	100	100	100	100	100	100	100	100	100	100	100	100	100	100
BR	Belarus	6	0	100	100	100	100	100	100	100	100	100	100	100	100	100	100	100	100	100	0
CZ	Czechia	37	100	100	100	100	100	100	100	100	100	100	100	100	100	100	100	100	100	100	100
DE	Germany	193	100	100	100	100	100	100	100	100	100	100	100	100	100	100	100	100	100	100	100
DK	Denmark	23	0	0	0	0	0	0	0	0	100	100	100	100	100	100	100	100	100	100	100
EE	Estonia	4	100	100	100	100	100	100	100	100	100	100	100	100	100	100	100	100	100	100	100
EL	Greece	21	100	100	100	100	100	100	100	100	100	100	100	100	100	100	100	100	100	100	100
ES	Spain	14	100	100	100	100	100	100	100	100	100	100	100	100	100	100	100	100	100	100	100
FI	Finland	9	100	100	100	100	100	100	100	100	100	100	100	100	100	100	100	100	100	100	100
FR	France	13	100	100	100	100	100	100	100	100	100	100	100	100	100	100	100	100	100	100	100
HR	Croatia	15	0	0	100	100	100	100	100	100	100	100	100	100	100	100	100	100	100	100	100
HU	Hungary	18	100	100	100	100	100	100	100	100	100	100	100	100	100	100	100	100	100	100	100
IS	Iceland	2	100	100	100	100	100	100	100	100	100	100	100	100	100	100	100	100	100	100	100
IT	Italy	30	0	100	100	100	100	100	100	100	100	100	100	100	100	100	100	100	100	100	100
LT	Lithuania	10	100	100	100	100	100	100	100	100	100	100	100	100	100	100	100	100	100	100	100
LV	Latvia	5	100	100	100	100	100	100	100	100	100	100	100	100	100	100	100	100	100	100	100
MK	North Macedonia	4	100	100	100	100	100	100	100	100	100	100	100	100	100	100	100	100	100	100	100
ML	Moldova	32	3.13	3.13	6.25	6.25	100	100	100	100	100	100	100	100	100	100	100	100	100	100	0
NL	Netherlands	8	100	100	100	100	100	100	100	100	100	100	100	100	100	100	100	100	100	100	100
PL	Poland	39	0	0	100	100	100	100	100	100	100	100	100	100	100	100	100	100	100	100	100
PT	Portugal	16	100	100	100	100	100	100	100	100	100	100	100	100	100	100	100	100	100	100	100
RO	Romania	40	100	100	100	100	100	100	100	100	100	100	100	100	100	100	100	100	100	100	100
RS	Serbia	22	0	0	0	0	0	0	0	0	0	0	0	100	100	100	100	100	100	100	100
RU	Russia	58	100	100	100	100	100	100	100	100	100	100	100	100	100	100	100	100	100	100	100
SE	Sweden	3	100	100	100	100	100	100	100	100	100	100	100	100	100	100	100	100	100	100	100
SI	Slovenia	4	0	0	0	100	100	100	100	100	100	100	100	100	100	100	100	100	100	100	100
SK	Slovakia	4	100	100	100	100	100	100	100	100	100	100	100	100	100	100	100	100	100	100	100
TR	Turkiye	12	0	0	0	0	0	0	0	0	0	0	100	100	100	100	100	100	100	100	100
UK	United Kingdom	18	0	0	100	100	100	100	100	100	100	100	100	100	100	100	100	100	100	100	100
UN	Ukraine	24	100	100	100	100	100	100	100	100	100	100	100	100	100	100	100	100	100	100	100

^3^.

Despite making use of Eurostat and National Statistics data, we are still affected by missing data for certain European countries. Of the 732 areas included in our analysis, 63.3% are represented by complete demographic time-series data. However, 37.7% are missing data for a demographic component, either fertility, mortality, or migration. Appendix 2 details the proportion of complete data by country. Imputation was performed by restructuring the demographic balancing equation, and has reduced the impact of missing data.

## Appendix 3

See Fig. 6.

Appendix 3 illustrates the trajectories of crude rates of demographic components in our five signatures, contextualising the annual changes in Fig. 2. Our signatures depict different contributions from demographic processes to population decline, and therefore we observe their distinct temporal trends here. In signatures *Steady Dual Deficit, Volatile Dual Deficit*, and *Migration Resurgence, *decreases in crude fertility rates and increases in crude mortality rates have produced a natural divergence. This acts to accelerate population declines and, in the signature *Migration Resurgence, *counteract contributions to population growth from positive net-migration increases. Conversely in signatures *Natural Reversal *and *Migration & Fertility Reversal*, we observe an opposite trend, with increases in crude fertility and decreases in crude mortality instigating a natural convergence. These demographic processes lead to a deceleration of population decline, albeit temporary as crude fertility rates fall in both signatures.

Although demographic trends ultimately differ in direction and timing across our signatures, we observe broad similarities in the demographic causes of population decline. In all signatures, fertility rates are outweighed by that of mortality consistently throughout our time series, emphasising the ubiquitous role of natural deficits in causing contemporary sub-national population declines. Furthermore, all signatures, except *Migration Resurgence*, depict dual causes of depopulation. Fertility-morality discrepancies and negative net-migration trends are observed. This indicates that contemporary population declines are most often driven by dual deficits, with depopulation reinforced by both natural change and out-migration processes. Additionally, Appendix 3 demonstrates the mirroring of functional shapes between crude net-migration and the overall crude rate of population change curves. The consistency of this pattern across all signatures encodes a regularity of the role of net-migration balances in shaping population decline trajectories. Net-migration balances, then, work to counterbalance, mitigate or exacerbate population declines. In this context, migration represents the principle differentiating demographic factor of population decline trajectories across sub-national European areas.

## Appendix 4

See Fig. ^7^.

To verify our k = 5 cluster solution, we explore the results of a k-medoid clustering algorithm under differing values of k. Here we present the results from a k = 4 and a k = 6 solutions. We observe the grouping of two distinct processes under a k = 4 solution, and the splitting of a homogeneous process under a k = 6 solution. The latter results in two very similar population decline processes, distinguished only by a greater contribution to population growth by positive net-migration from the period 2005–2008 (clusters 2 and 6 above). These alternative cluster solutions provide credence to our k = 5 solution, as each cluster depicts a distinct set of demographic processes underpinning population decline.

## Appendix 5

See Fig. 8.

In accordance with the NbClust package (Charrad et al., 2014), we empirically evaluate the optimal value of K in our clustering procedure. In total, 30 indices are tested, with the majority determining that a K = 5 solution is appropriate. We consider this information, along with a visual inspection of alternative K solutions (Appendix 4) to reach a decision.

## Appendix 6

See Table

**Table 4:** Results of mfPCA analysis

2 multivariate functional principal components estimated with 3 elements		
	PC 1	PC 2
Eigen value	0.4605	0.0297
Proportion of variance explained	0.9393	0.0606
Cumualative proportion	0.9393	1.000

^4^.

As part of the third stage of our analysis, we conduct a multivariate functional principal component analysis. We use this to produce mfPCA scores, which represent the unique contributions of crude fertility, mortality and net-migration to population change for each area (n = 732) from 2000–2018. Appendix 6 demonstrates the initial results of this stage, demonstrating how just two principal components explain 100% of the variance of input variables. This could be explained by the simple structure of our input data, the few number of dimensions (3 – fertility, mortality, and net-migration) or the existence of strong correlations between them.

## Footnotes

1 This does not mean that this component has no contribution to population decline; for that specific period conditions are the same and so there is no change to that specific year.

## References

[CR1] Aldridge, R. W., Nellums, L. B., Bartlett, S., Barr, A. L., Patel, P., Burns, R., Sally Hargreaves, J., Miranda, J., Tollman, S., Friedland, J. S., & Abubakar, I. (2018). Global patterns of mortality in international migrants: A systematic review and meta-analysis. *The Lancet,**392*(10164), 2553–2566. 10.1016/S0140-6736(18)32781-8 10.1016/S0140-6736(18)32781-8 PMC6294735 30528484

[CR2] Beaujouan, E. (2020). Latest-late fertility? Decline and resurgence of late parenthood across the low-fertility countries. *Population and Development Review,**46*(2), 219–247. 10.1111/padr.12334 32733116 10.1111/padr.12334 PMC7384131

[CR3] Bell, M., Charles-Edwards, E., Kupiszewska, D., Kupiszewski, M., Stillwell, J., & Zhu, Y. (2014). Internal migration data around the world: Assessing contemporary practice. *Population, Space and Place,**21*(1), 1–17. 10.1002/psp.1848

[CR300] Bellman, B., Spielman, S. E., & Franklin, R. S. (2018). Local population change and variations in racial integration in the United States, 2000-2010. *International Regional Science Review, 41*(2), 233–255. 10.1177/0160017616665669 PMC5922766 29713109

[CR301] Bock, B., & Haartsen, T. (2021). Who is afraid of population decline? The struggle of keeping rural depopulation on the Dutch agenda. *AGER: Journal of Depopulation and Rural Development Studies, 33*, 35–56.

[CR4] Bongaarts, J. (1978). A framework for analyzing the proximate determinants of fertility. *Population and Development Review,**4*(1), 105. 10.2307/1972149

[CR100] Bongaarts, J. (2009). Human population growth and the demographic transition. *Philosophical Transactions of the Royal Society B: Biological Sciences,**364*(1532), 2985–2990 10.1098/rstb.2009.0137 10.1098/rstb.2009.0137 PMC2781829 19770150

[CR5] Bremberg, S. (2020). Rural-urban mortality inequalities in four Nordic welfare states. *Scandinavian Journal of Public Health,**48*(8), 791–793. 10.1177/1403494820921684 32456534 10.1177/1403494820921684 PMC7678336

[CR6] Campisi, N., Kulu, H., Mikolai, J., Klüsener, S., & Myrskylä, M. (2020). Spatial variation in fertility across Europe: Patterns and determinants. *Population, Space and Place,**26*(4), e2308. 10.1002/psp.2308

[CR7] Canudas-Romo, V. (2003). *Decomposition methods in demography*. Rozenberg Publishers.

[CR9] Charrad, M., Ghazzali, N., Boiteau, V., & Niknafs, A. (2014). NbClust: An R package for determining the relevant number of clusters in a data set. *Journal of Statistical Software*. 10.18637/jss.v061.i06

[CR10] Cheng, X., Yang, Y., Schwebel, D. C., Liu, Z., Li, L., Cheng, P., Ning, P., & Hu, G. (2020). Population ageing and mortality during 1990–2017: A global decomposition analysis. *PLOS Medicine,**17*(6), e1003138. 10.1371/journal.pmed.1003138 32511229 10.1371/journal.pmed.1003138 PMC7279585

[CR12] Coleman, D., & Rowthorn, R. (2011). Who’s afraid of population decline? A critical examination of its consequences. *Population and Development Review,**37*(s1), 217–248. 10.1111/j.1728-4457.2011.00385.x 21280372 10.1111/j.1728-4457.2011.00385.x

[CR13] Cooke, L. P. (2009). Gender equity and fertility in Italy and Spain. *Journal of Social Policy,**38*(1), 123–140. 10.1017/S0047279408002584

[CR14] Cutler, D., Deaton, A., & Lleras-Muney, A. (2006). The determinants of mortality. *Journal of Economic Perspectives,**20*(3), 97–120. 10.1257/jep.20.3.97

[CR15] Denisenko, M., Mkrtchyan, N., & Chudinovskikh, O. (2020). Permanent migration in the post-Soviet countries. In Mikhail Denisenko, Salvatore Strozza, & Matthew Light (Eds.), *Migration from the newly independent states: 25 years after the collapse of the USSR* (pp. 23–53). Springer. 10.1007/978-3-030-36075-7_3

[CR16] Domnich, A., Panatto, D., Gasparini, R., & Amicizia, D. (2012). The “healthy immigrant” effect: Does it exist in Europe today? *Italian Journal of Public Health*. 10.2427/7532

[CR17] Ebeling, M., Rau, R., Sander, N., Kibele, E., & Klüsener, S. (2022). Urban–rural disparities in old-age mortality vary systematically with age: Evidence from Germany and England & Wales. *Public Health,**205*, 102–109. 10.1016/j.puhe.2022.01.023 35276525 10.1016/j.puhe.2022.01.023

[CR999] Elshof, H., Van Wissen, L., & Mulder, C. H. (2014). The self-reinforcing effects of population decline: An analysis of differences in moving behaviour between rural neighbourhoods with declining and stable populations. *Journal of Rural Studies*, *36*, 285–299.

[CR18] European Parliament, Committee on Regional Development. (2021). Report on reversing demographic trends in EU regions using cohesion policy instruments (2020/2039(INI)). https://www.europarl.europa.eu/doceo/document/A-9-2021-0061_EN.html#:~:text=Urges%20Member%20States%20and%20regional,35.

[CR19] Eurostat. (2022). Migration and migrant population statistics. https://ec.europa.eu/eurostat/statistics-explained/index.php?title=Migration_and_migrant_population_statistics#Migrant_population:_23.8_million_non-EU_citizens_living_in_the_EU_on_1_January_2022

[CR20] Fihel, A., Janicka, A., & Kloc-Nowak, W. (2018). The direct and indirect impact of international migration on the population ageing process: A formal analysis and its application to Poland. *Demographic Research,**38*, 1303–1338. 10.4054/DemRes.2018.38.43

[CR21] Fihel, A., & Okólski, M. (2019). Population decline in formerly communist countries of the European Union. *Population & Societies,**6*, 1–4. 10.3917/popsoc.567.0001

[CR22] Fisher, K., & Robinson, J. (2011). Daily life in 23 countries. *Social Indicators Research,**101*(2), 295–304. 10.1007/s11205-010-9650-3 21475388 10.1007/s11205-010-9650-3 PMC3046343

[CR23] Franklin, R. S. (2019). The demographic burden of population loss in US cities, 2000–2010. *Journal of Geographical Systems,**23*(2), 209–230. 10.1007/s10109-019-00303-4

[CR24] Franklin, R. S. (2020). I come to bury (population) growth, not to praise it. *Spatial Economic Analysis,**15*(4), 359–373. 10.1080/17421772.2020.1802056 33727949 10.1080/17421772.2020.1802056 PMC7959099

[CR25] Gompertz, B. (1825). XXIV. On the nature of the function expressive of the law of human mortality, and on a new mode of determining the value of life contingencies. In a letter to Francis Baily, Esq. FRS &c. *Philosophical transactions of the Royal Society of London*, (115), 513–583. http://www.jstor.org/stable/107756

[CR26] González-Leonardo, M., López-Gay, A., Newsham, N., Recaño, J., & Rowe, F. (2022). Understanding patterns of internal migration during the COVID-19 pandemic in Spain. *Population, Space and Place,**28*(6), e2578. 10.1002/psp.2578 35942493 10.1002/psp.2578 PMC9350359

[CR27] González-Leonardo, M., Newsham, N., & Rowe, F. (2023). Understanding population decline trajectories in Spain using sequence analysis. *Geographical Analysis*. 10.1111/gean.12357

[CR28] Grigoriev, P., Shkolnikov, V., Andreev, E., Jasilionis, D., Jdanov, D., Meslé, F., & Vallin, J. (2010). Mortality in Belarus, Lithuania, and Russia: Divergence in recent trends and possible explanations. *European Journal of Population / Revue Européenne de Démographie,**26*(3), 245–274. 10.1007/s10680-010-9210-1

[CR29] Gupta, P. D. (1993). Standardization and decomposition of rates: A user's manual (No. 186). US Department of Commerce, Economics and Statistics Administration, Bureau of the Census. C

[CR30] Haase, A., Bernt, M., Großmann, K., Mykhnenko, V., & Rink, D. (2016). Varieties of shrinkage in European cities. *European Urban and Regional Studies,**23*(1), 86–102. 10.1177/0969776413481985

[CR31] Happ, C., & Greven, S. (2018). Multivariate functional principal component analysis for data observed on different (dimensional) domains. *Journal of the American Statistical Association,**113*(522), 649–659. 10.1080/01621459.2016.1273115

[CR32] Happ-Kurz, C. (2020). Object-oriented software for functional data. *Journal of Statistical Software*. 10.18637/jss.v093.i05

[CR33] Hartt, M. D. (2018). How cities shrink: Complex pathways to population decline. *Cities,**75*, 38–49. 10.1016/j.cities.2016.12.005

[CR34] Hoekveld, J. J. (2012). Time-space relations and the differences between shrinking regions. *Built Environment,**38*(2), 179–195. 10.2148/benv.38.2.179

[CR35] Horiuchi, S. (1992). Stagnation in the decline of the world population growth rate during the 1980s. *Science,**257*(5071), 761–765. 10.1126/science.1496396 1496396 10.1126/science.1496396

[CR36] Horiuchi, S. (1995). The cohort approach to population growth: a retrospective decomposition of growth rates for Sweden. *Population Studies,**49*(1), 147–163. 10.1080/0032472031000148296

[CR995] Hospers, G. J., & Reverda, N. (2015). *Managing population decline in Europe’s urban and rural areas* (pp. 10–14). Springer.

[CR37] Hrzic, R., Vogt, T., Janssen, F., & Brand, H. (2020). Mortality convergence in the enlarged European Union: A systematic literature review. *European Journal of Public Health,**30*(6), 1108–1115. 10.1093/eurpub/ckaa038 32206793 10.1093/eurpub/ckaa038 PMC7733049

[CR38] Hyndman, R. J., & Booth, H. (2008). Stochastic population forecasts using functional data models for mortality, fertility and migration. *International Journal of Forecasting,**24*(3), 323–342. 10.1016/j.ijforecast.2008.02.009

[CR39] Janssen, F., Trias-Llimós, S., & Kunst, A. E. (2021). The combined impact of smoking, obesity and alcohol on life-expectancy trends in Europe. *International Journal of Epidemiology,**50*(3), 931–941. 10.1093/ije/dyaa273 10.1093/ije/dyaa273 PMC8271206 33432332

[CR40] Johnson, K. M., Field, L. M., & Poston, D. L., Jr. (2015). More deaths than births: Subnational natural decrease in Europe and the United States. *Population and Development Review,**41*(4), 651–680. 10.1111/j.1728-4457.2015.00089.x

[CR41] Johnson, K. M., & Lichter, D. T. (2019). Rural depopulation: growth and decline processes over the past century. *Rural Sociology,**84*(1), 3–27. 10.1111/ruso.12266

[CR42] Jung, P. H., & Song, J. (2022). Multivariate neighborhood trajectory analysis: An exploration of the functional data analysis approach. *Geographical Analysis,**54*(4), 789–819. 10.1111/gean.12298

[CR43] Karanikolos, M., Adany, R., & McKee, M. (2017). The epidemiological transition in Eastern and Western Europe: A historic natural experiment. *European Journal of Public Health,**27*, 4–8. 10.1093/eurpub/ckx158 10.1093/eurpub/ckx158 29028237

[CR44] King, R., & Okólski, M. (2018). Diverse, fragile and fragmented: The new map of European migration. *Central and Eastern European Migration Review*, *Advance online publication*, 1–24. 10.17467/ceemr.2018.18

[CR45] Kirk, D. (1996). Demographic transition theory. *Population Studies,**50*(3), 361–387. 11618374 10.1080/0032472031000149536

[CR47] Kohler, H. P., Billari, F. C., & Ortega, J. A. (2006). Low fertility in Europe: Causes, implications and policy options. In F. R. Harris (Ed.), *The baby bust: Who will do the work? Who will pay the taxes?* (pp. 48–109). Rowman & Littlefield Publishers.

[CR48] Kulu, H. (2013). Why do fertility levels vary between urban and rural areas? *Regional Studies,**47*(6), 895–912. 10.1080/00343404.2011.581276

[CR49] Kulu, H., Hannemann, T., Pailhé, A., Neels, K., Krapf, S., González-Ferrer, A., & Andersson, G. (2017). Fertility by birth order among the descendants of immigrants in selected European countries. *Population and Development Review*. 10.1111/padr.12037

[CR50] Lesthaeghe, R., & Willems, P. (1999). Is low fertility a temporary phenomenon in the European Union? *Population and Development Review,**25*(2), 211–228. 10.1111/j.1728-4457.1999.00211.x

[CR51] Li, Y., Westlund, H., & Liu, Y. (2019). Why some rural areas decline while some others not: An overview of rural evolution in the world. *Journal of Rural Studies,**68*, 135–143. 10.1016/j.jrurstud.2019.03.003

[CR52] Lutz, W., & Gailey, N. (2020). *Depopulation as a policy challenge in the context of global demographic trends*. UNDP Serbia: Human Development Series.

[CR53] Lycan, R., & Rynerson, C. (2013). Senior shedding: Mortality and migration of seniors create vacancies for gentrifying neighborhoods. Presentation to American Association of Geographers, Los Angeles, CA, April 2013.

[CR54] Martí-Henneberg, J. (2005). Empirical evidence of regional population concentration in Europe, 1870–2000. *Population, Space and Place,**11*(4), 269–281. 10.1002/psp.373

[CR55] Matysiak, A., Sobotka, T., & Vignoli, D. (2021). The great recession and fertility in Europe: A sub-national analysis. *European Journal of Population,**37*(1), 29–64. 10.1007/s10680-020-09556-y 33597835 10.1007/s10680-020-09556-y PMC7864853

[CR56] McDonald, P. (2000). Gender equity in theories of fertility transition. *Population and Development Review,**26*(3), 427–439. 10.1111/j.1728-4457.2000.00427.x

[CR57] Meslé, F., Vallin, J., & Obadia, Y. (2002). Mortality in Europe: The divergence between east and west. *Population,**57*(1), 157–197. 10.2307/3246630

[CR58] Myrskylä, M., Kohler, H. P., & Billari, F. (2011). High development and fertility: Fertility at older reproductive ages and gender equality explain the positive link. Max Planck Institute for Demographic Research, Rostock, Germany

[CR59] Newsham, N., & Rowe, F. (2021). Projecting the demographic impact of Syrian migration in a rapidly ageing society Germany. *Journal of Geographical Systems,**23*(2), 231–261. 10.1007/s10109-018-00290-y

[CR60] Newsham, N., & Rowe, F. (2022). Understanding trajectories of population decline across rural and urban Europe: A sequence analysis. *Population, Space and Place,**29*(3), e2630. 10.1002/psp.2630

[CR61] Neyer, G., & Andersson, G. (2008). Consequences of family policies on childbearing behavior: Effects or artifacts? *Population and Development Review,**34*(4), 699–724. 10.1111/j.1728-4457.2008.00246.x

[CR62] Patias, N., Rowe, F., & Arribas-Bel, D. (2023). Local urban attributes defining ethnically segregated areas across English cities: A multilevel approach. *Cities,**132*, 103967. 10.1016/j.cities.2022.103967

[CR63] Preston, S. H., Heuveline, P., & Guillot, M. (2000). *Demography: Measuring and modeling population processes*. Blackwell Publishers Ltd.

[CR64] Pullum, T.W., & Jadhav, A. (2021). A decomposition of sources of change in population size and median age, 1970–2020. DHS Working Papers No. 178. Rockville, Maryland, USA: ICF.

[CR65] Pužulis, A., & Kūle, L. (2016). Shrinking of rural territories in Latvia. *European Integration Studies,**10*, 90–105. 10.5755/j01.eis.0.10.14988

[CR66] Raleigh, V. S. (2019). Trends in life expectancy in EU and other OECD countries: Why are improvements slowing? 10.1787/18152015

[CR68] Ramsay, J. O., & Dalzell, C. (1991). Some tools for functional data analysis. *Journal of the Royal Statistical Society Series B: Statistical Methodology,**53*(3), 539–561. 10.1111/j.2517-6161.1991.tb01844.x

[CR70] Ramsay, J. O., & Silverman, B. W. (2005). Principal components analysis for functional data. *Functional Data Analysis*, 147–172.

[CR71] Raugze, I., Daly, G., & van Herwijnen, M. (2017). Shrinking rural regions in Europe. Towards smart and innovative approaches to regional development challenges in depopulating rural regions. ESPON EGTC Policy Brief: 15pp. Luxembourg: ESPON EGTC

[CR72] Reher, D. S. (1998). Family ties in Western Europe: persistent contrasts. *Population and Development Review,**24*(2), 203–234. 10.2307/2807972

[CR73] Reher, D. (2005). Long-term population decline, past and future. In *International Union for the scientific Study of Population. XXV International Population Conference*. Session (Vol. 94).

[CR99] Reher, D. (2007) Towards long-term population decline: A discussion of relevant issues. *European Journal of Population / Revue Européenne de Démographie,**23*(2), 189–207. 10.1007/s10680-007-9120-z

[CR74] Reynaud, C., & Miccoli, S. (2018). Depopulation and the aging population: The relationship in Italian municipalities. *Sustainability,**10*(4), 1004. 10.3390/su10041004

[CR75] Reynaud, C., Miccoli, S., Benassi, F., Naccarato, A., & Salvati, L. (2020). Unravelling a demographic ‘Mosaic’: Spatial patterns and contextual factors of depopulation in Italian municipalities, 1981–2011. *Ecological Indicators,**115*, 106356. 10.1016/j.ecolind.2020.106356

[CR76] Rodríguez-Pose, A., & Ketterer, T. D. (2012). Do local amenities affect the appeal of regions in Europe for migrants? *Journal of Regional Science,**52*(4), 535–561. 10.1111/j.1467-9787.2012.00779.x

[CR77] Rodríguez-Vignoli, J., & Rowe, F. (2018). How is internal migration reshaping metropolitan populations in Latin America? A new method and new evidence. *Population Studies,**72*(2), 253–273. 10.1080/00324728.2017.1416155 29380654 10.1080/00324728.2017.1416155

[CR78] Rogers, A., & Castro, L. J. (1981). *Model migration schedules*. Laxenburg: International Institute for Applied System Analysis.

[CR79] Romaniuk, A., & Gladun, O. (2015). Demographic trends in Ukraine: Past, present, and future. *Population and Development Review,**41*(2), 315–337. 10.1111/j.1728-4457.2015.00049.x

[CR80] Rowe, F., Bell, M., Bernard, A., Charles-Edwards, E., & Ueffing, P. (2019). Impact of internal migration on population redistribution in Europe: Urbanisation, counterurbanisation or spatial equilibrium? *Comparative Population Studies*. 10.12765/CPoS-2019-18

[CR81] Shang, H. L., Smith, P. W., Bijak, J., & Wiśniowski, A. (2016). A multilevel functional data method for forecasting population, with an application to the United Kingdom. *International Journal of Forecasting,**32*(3), 629–649. 10.1016/j.ijforecast.2015.10.002

[CR82] Shcherbakova, E. M. (2022). Population dynamics in Russia in the context of global trends. *Studies on Russian Economic Development,**33*(4), 409–421. 10.1134/S1075700722040098 35911060 10.1134/S1075700722040098 PMC9321303

[CR83] Sobotka, T. (2004). *Postponement of childbearing and low fertility in Europe*. Dutch University Press.

[CR84] Sobotka, T. (2008). Overview chapter 7: The rising importance of migrants for childbearing in Europe. *Demographic Research,**19*, 225–248. 10.4054/DemRes.2008.19.9

[CR85] Sobotka, T., Skirbekk, V., & Philipov, D. (2011). Economic recession and fertility in the developed world. *Population and Development Review,**37*(2), 267–306. 10.1111/j.1728-4457.2011.00411.x 22066128 10.1111/j.1728-4457.2011.00411.x

[CR86] Sobotka, T., Matysiak, A., & Brzozowska, Z. (2019). Policy responses to low fertility: How effective are they*? United Nations Population Fund.*

[CR87] Sobotka, T., & Fürnkranz-Prskawetz, A. (2020). Demographic change in Central, Eastern and Southeastern Europe: Trends, determinants and challenges. In Robert Holzmann, Doris Ritzberger-Grünwald, & Helene Schuberth (Eds.), *30 years of transition in Europe: Looking back and looking beyond in CESEE countries. *Edward Elgar Publishing. 10.4337/9781839109508.00027

[CR88] Sobotka, T., Jasilioniene, A., Zeman, K., Winkler-Dworak, M., Brzozowska, Z., Galarza, A. A., & Jdanov, D. (2022). From bust to boom? Birth and fertility responses to the COVID-19 pandemic. *SocArXiv*. 10.31235/osf.io/87acb

[CR89] Thomas, J., Rowe, F., & Lin, E. S. (2022). Declining fertility in Taiwan: The deterring impact of housework imbalance. *Asian Population Studies,**19*(3), 270–288. 10.1080/17441730.2022.2035555

[CR90] United Nations, Department of Economic and Social Affairs, Population Division. (2022).* World Population prospects 2022: Summary of Results*. UN DESA/POP/2022/TR/NO. 3.

[CR91] United Nations Economic Commission for Europe. (2017). *Older persons in rural and remote areas*. UNECE Policy Brief on Ageing No. 18. Available: https://unece.org/policy-briefs

[CR92] Vallin, J., & Meslé, F. (2004). Convergences and divergences in mortality: A new approach of health transition. *Demographic Research,**2*, 11–44. 10.4054/DemRes.2004.S2.2

[CR888] Van Dalen, H. P., & Henkens, K. (2011). Who fears and who welcomes population decline? *Demographic Research, 25*, 437–464.

[CR93] Vaupel, J. W., & Canudas-Romo, V. (2002). Decomposing demographic change into direct vs compositional components. *Demographic Research,**7*, 1–14. 10.4054/DemRes.2002.7.1

[CR777] Vignoli, D., Guetto, R., Bazzani, G., Pirani, E., & Minello, A. (2020). A reflection on economic uncertainty and fertility in Europe: The narrative framework. *Genus, 76*, 1–27. 10.1186/s41118-020-00094-3 PMC7480209 32921800

[CR94] Viñas, C. D. (2019). Depopulation processes in European rural areas: A case study of Cantabria (Spain). *European Countryside,**11*(3), 341–369. 10.2478/euco-2019-0021

[CR95] Wang, H., Paulson, K. R., Pease, S. A., Watson, S., Comfort, H., Zheng, P., & Murray, C. J. (2022). Estimating excess mortality due to the COVID-19 pandemic: A systematic analysis of COVID-19-related mortality, 2020–21. *The Lancet,**399*(10334), 1513–1536. 10.1016/S0140-6736(21)02796-3 PMC8912932 35279232

[CR96] Wolff, M., & Wiechmann, T. (2018). Urban growth and decline: Europe’s shrinking cities in a comparative perspective 1990–2010. *European Urban and Regional Studies,**25*(2), 122–139. 10.1177/0969776417694680

[CR97] Zagheni, E., Garimella, V. R. K., Weber, I., & State, B. (2014). Inferring international and internal migration patterns from Twitter data. In *Proceedings of the 23rd international conference on world wide web*, (pp. 439–444). 10.1145/2567948.2576930

[CR98] Zelinsky, W. (1971). The hypothesis of the mobility transition. *Geographical Review,**61*(2), 219. 10.2307/213996 10.1111/gere.12310 PMC7269166 32494088

